# Loss of VHL-mediated pRb regulation promotes clear cell renal cell carcinoma

**DOI:** 10.1038/s41419-025-07623-y

**Published:** 2025-04-16

**Authors:** Mercy Akuma, Minjun Kim, Chenxuan Zhu, Ellis Wiljer, Antoine Gaudreau-Lapierre, Leshan D. Patterson, Lars Egevad, Simon Tanguay, Laura Trinkle-Mulcahy, William L. Stanford, Yasser Riazalhosseini, Ryan C. Russell

**Affiliations:** 1 Department of Cellular and Molecular Medicine, University of Ottawa, Ottawa, ON K1H 8M5 Canada; 2 Department of Human Genetics, McGill University, Montreal, QC H3A 0G1 Canada; 3 Victor Phillip Dahdaleh Institute of Genomic Medicine, McGill University, Montreal, QC, Canada; 4 Ottawa Hospital Research Institute (OHRI), Ottawa, ON K1H 8L6 Canada; 5 Department of Biochemistry, Microbiology and Immunology, University of Ottawa, Ottawa, ON K1H 8M5 Canada; 6 Ottawa Institute of Systems Biology, University of Ottawa, Ottawa, ON, Canada; 7 Department of Science, University of Waterloo, Waterloo, ON N2L 3G1 Canada; 8 Department of Oncology-Pathology, Karolinska Institutet, Stockholm, Sweden; 9 Department of Surgery, Division of Urology, McGill University, Montreal, QC, Canada; 10 Department of Biochemistry, University of Toronto, Toronto, ON, Canada; 11 University of Ottawa Centre for Infection, Immunity and Inflammation, Ottawa, ON Canada

**Keywords:** Cancer, Ubiquitin ligases

## Abstract

The von Hippel-Lindau (VHL) tumor suppressor is a substrate-defining component of E3 ubiquitin ligase complexes that target cellular substrates for proteasome-mediated degradation. VHL inactivation by mutation or transcriptional silencing is observed in most sporadic cases of clear cell renal cell carcinoma (ccRCC). VHL loss in ccRCC leads to constitutive stabilization of E3 ligase substrates, including hypoxia inducible factor α (HIFα). HIFα stabilization upon VHL loss is known to contribute to ccRCC development through transactivation of hypoxia-responsive genes. HIF-independent VHL targets have been implicated in oncogenesis, although those mechanisms are less well-defined than for HIFα. Using proximity labeling to identify proteasomal-sensitive VHL interactors, we identified retinoblastoma protein (pRb) as a novel substrate of VHL. Mechanistically, VHL interacts with pRb in a proteasomal-sensitive manner, promoting its ubiquitin-mediated degradation. Concordantly, VHL-inactivation results in pRb hyperstabilization. Functionally, loss of pRb in ccRCC led to increased cell death, transcriptional changes, and loss of oncogenic properties in vitro and in vivo. We also show that downstream transcriptional changes induced by pRb hyperstabilization may contribute to ccRCC tumor development. Together, our findings reveal a novel VHL-related pathway which can be therapeutically targeted to inhibit ccRCC tumor development.

## Introduction

Von Hippel-Lindau (VHL) disease is a familial autosomal dominant disorder characterized by the predisposition to develop highly vascularized and glycolytic tumors in multiple organs [1]. VHL disease conforms to the Knudson’s ‘two-hit’ model of oncogenesis [2–4]. Germline mutations in VHL result in a defective allele and this represents the first ‘hit’. The second ‘hit’ is a somatic event and oncogenic transformation on the second allele, often a deletion, leading to loss of heterozygosity at the VHL locus. The clinical manifestations of VHL disease include retinal and central nervous system hemangioblastomas, clear cell renal cell carcinoma (ccRCC), pheochromocytomas, pancreatic neuroendocrine tumors, and endolymphatic sac tumors (ELSTs) [1, 5–10]. However, the leading cause of mortality in patients with VHL disease is metastatic ccRCC [11].

Biallelic VHL inactivation has also been observed in approximately 70% of sporadic ccRCCs [12, 13]. The best characterized function of VHL relates to its role as the substrate recognition component of an E3 ubiquitin ligase complex that also contains Elongin B, Elongin C, Cullin-2 and Rbx1 (called VCB-CUL2 complex) [14–16]. The VCB-CUL2 complex promotes the ubiquitination and proteasomal degradation of the hypoxia inducible factor α (HIFα) under normoxia [17, 18]. VHL inactivation in ccRCC leads to constitutive HIFα stabilization, irrespective of oxygen levels [19]. HIFα activates expression of genes that promote tumor adaptation and development, linking HIF stabilization to tumorigenesis [20–25]. Downregulation of HIF2α was shown to be sufficient to suppress tumorigenesis in VHL-defective ccRCC cells, while stable expression of VHL-resistant HIF2α can promote tumor growth in ccRCC cells reconstituted with VHL [26, 27]. As such, significant effort has been put into pharmacologically targeting HIF or HIF-related pathways [28–34]. However, the most significant therapeutic progress has been a result of combination treatments involving immune-oncologic drugs [35–37]. Despite improved response with the introduction of immune-oncologic drugs, the majority of patients with metastatic ccRCC are still resistant to all current therapeutic options [38, 39]. Therefore, identifying novel pathways underlying ccRCC is needed to establish new therapeutic targets.

There are several ‘non-HIF’ targets of VHL such as Epidermal Growth Factor Receptor (EGFR), Zinc finger and homeobox 2 (ZHX2), and β_2_-adrenergic receptor (β_2_AR) that are involved in signal transduction [40–42]. Other targets include atypical protein kinase C (aPKC), Sprouty2 (Spry2), and Scm-like with four malignant brain tumor domains 1 (SFMBT1), which regulate cellular differentiation [43–45]. These HIF-independent VHL substrates may also be involved in cancer development. For example, ZHX2 may promote ccRCC oncogenesis via activation of the Nuclear factor kappa B (NF-κB) pathway [41]. Also, SFMBT1, a key regulator of epithelial-to-mesenchymal transition, was shown to be upregulated in ccRCC and contribute to tumor growth [45, 46]. In support for the biological relevance of HIF-independent VHL targets, a synthetic mutant of VHL that retains HIF degradation, but is deficient in the regulation of some HIF-independent targets, was found to be incapable of suppressing ccRCC tumorigenesis [47, 48]. These findings highlight the potential contributions of non-HIF VHL substrates to tumor development. Interestingly, conditional knockout of pRb was shown to be synthetically lethal with VHL loss in the retina, another tissue impacted in VHL kindred [49]. The increase in cell death in *Vhl/Rb1* double knockout retinas was also accompanied by increased angiogenesis in unaffected cells, indicating an abnormal response to the pseudohypoxia caused by *Vhl* loss. Dual inhibition of CDK4 and 6 was found to be synthetically lethal with VHL loss in a HIF-independent manner, although whether this effect was dependent on changes in pRb phosphorylation was not addressed [50]. Together, these studies indicate that VHL and pRb may coordinate cell death in diverse cell types and tissues.

pRb is involved in the regulation of cellular processes such as G1-S transition, DNA damage checkpoint, cell cycle exit, and cellular differentiation [51–54]. When hypophosphorylated, pRb interacts with the E2F transcription factors, inhibiting the transcription of genes required for S-phase entry [55–59]. However, prior to cell division, pRb becomes hyperphosphorylated, inhibiting its ability to sequester E2Fs and thereby promoting cell cycle. Inactivation of pRb can lead to loss of cell cycle checkpoints, which contribute to pRb’s tumor suppressor function. It is therefore common for tumor cells to inactivate pRb’s growth suppressive function by exploiting pathways that regulate pRb phosphorylation [60, 61]. For example, cyclin D1 overexpression observed in ccRCC cells results in pRb hyperphosphorylation, which supports abnormal proliferation in cells deprived of growth factors [62].

pRb has also been shown to regulate cell-cycle-independent pathways such as inflammation, autophagy, apoptosis, metabolism and stemness [63–68], which have the potential to contribute to oncogenesis. For instance, pRb is described to have anti-apoptotic functions and its inactivation is associated with increased levels of apoptosis [69–73]. In colorectal cancer cells, antisense (AS) oligodeoxynucleotide targeting of pRb mRNA led to growth inhibition and induction of apoptosis, suggesting that pRb may protect against apoptosis [73]. pRb has also been shown to suppress angiogenesis and autophagic cell death under hypoxia [49, 74]. Therefore, pRb may promote oncogenesis in some contexts. Accordingly, pRb overexpression has been observed in multiple cancer types including familial adrenocortical carcinomas, pancreatic adenocarcinomas, and colorectal carcinomas [75–77]. Interestingly, overexpression of phosphorylation-resistant constitutively active pRb in the mammary gland led to the development of mammary adenocarcinoma [78]. pRb also antagonizes E2F1 repression of beta-catenin transcription to promote colorectal tumor growth [79]. Together these studies highlight a potential oncogenic function of pRb in diverse tumor types.

In this study, we characterize pRb as a novel substrate of VHL, which is targeted for ubiquitin-dependent degradation in a VHL-sensitive manner. We found that pRb was upregulated in ccRCC and that high pRb protein expression is associated with increased tumorigenic potential of ccRCC cells. We further demonstrate that pRb inhibits apoptosis in ccRCC cells and that this function may be via regulation of downstream transcriptional targets including Ski/Dach domain-containing protein 1 (SKIDA1), a minimally characterized protein. Overall, our findings highlight the proteasomal-sensitive regulation of pRb by VHL and the potential implications of VHL-pRb dysregulation on hypoxia-mediated cell death in ccRCC tumors. Importantly, we present a novel VHL-related pathway which can be therapeutically targeted to inhibit ccRCC tumor development.

## Results

### pRb binds VHL in a proteasomal-sensitive manner

**Fig. 1:**
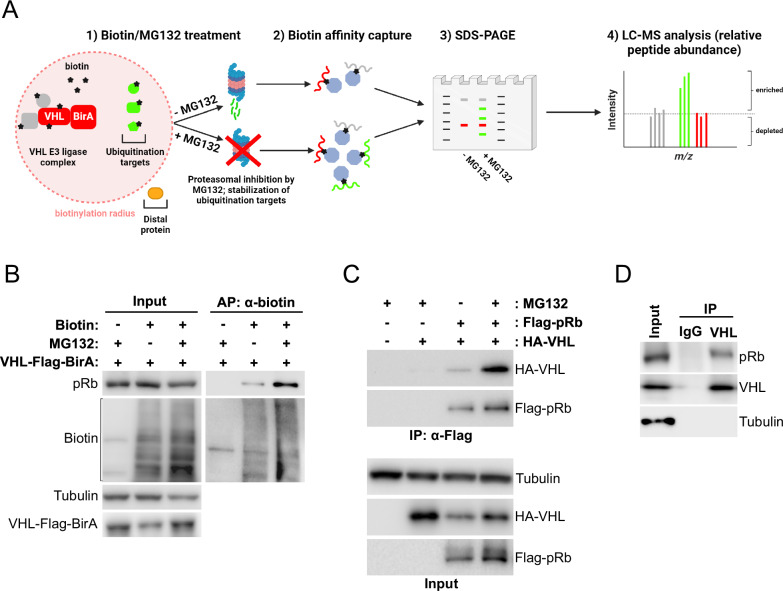
Proximity ligation analysis of VHL reveals pRb as a proteasome-sensitive VHL interactor. **A** Schematic diagram illustrating BioID assay used to identify VHL interacting proteins. Figure was created with BioRender. In the experimental condition, 10 µM MG132 was added for 4 h, followed by 50 µM biotin for 1 h. In the control condition, 10 µM DMSO vehicle was added instead of MG132. Biotinylated proteins were captured using streptavidin-conjugated beads and resolved on SDS-PAGE gel. Gel chunks were then extracted separately from control and experimental lanes, trypsin digested and analyzed by mass spectrometry. **B** Affinity purification of biotinylated proteins using streptavidin-conjugated beads from HEK293A cells transfected with VHL-Flag-BirA plasmid and treated with 10 µM MG132 and 50 µM biotin as indicated for 4 h and 1 h, respectively. **C** Immunoprecipitation of Flag-tagged pRb from HEK293A cells transfected with indicated plasmids and treated with 10 µM MG132 as indicated for 4 h. **D** Immunoprecipitation of endogenous VHL from HEK293A cells treated with 10 µM MG132 for 4 h. An IgG1 isotype control was included. MG132-treated HEK293A lysate was split equally between IgG1 and VHL pulldown conditions.

The interaction between VHL and HIF is stabilized in the presence of proteasome inhibitors [80–82]. To identify novel proteasome-sensitive VHL substrates that might contribute to disease, we performed a proximity-dependent biotin identification (BioID) assay using HEK293A cells expressing VHL fused to the biotin ligase BirA. Cells were pre-treated with the proteasome inhibitor MG132 or vehicle control for 4 hours, followed by 1 hour of biotin addition. MG132 stabilizes ubiquitinated proteins marked for degradation by blocking the proteolytic activity of the 26S proteasome complex and this is known to stabilize the interaction of VHL and its targets, including HIFα [19]. Biotinylated proteins were captured using streptavidin-conjugated beads and resolved on an SDS-PAGE gel. Proteins were visualized following a Coomassie stain. Protein-containing gel slices were then excised, trypsin-digested, and analyzed by liquid chromatography-mass spectrometry (LC-MS) (Fig. 1A). LC-MS analysis revealed several proteins that were enriched in MG132-treated cells compared to vehicle control. Interestingly, the tumor suppressor pRb was one of the proteins enriched following MG132 treatment, raising the possibility that pRb is a proteasomal target of VHL. Despite the apparent paradox of one tumor suppressor degrading another, the prior data describing the synthetic lethality of *Vhl* and *Rb1* loss [49], and pRb’s role in modulating hypoxic cell death [74], led us to validate pRb as our top hit.

To validate our mass spectrometry results, we first sought to confirm the enrichment of pRb in samples preferentially biotinylated by VHL-BirA in MG132-treated cells. Cells were transfected with VHL-BirA and treated with biotin and MG132 or vehicle as described above. Biotinylated proteins were pulled down with streptavidin beads and eluted proteins were resolved by SDS-PAGE. Immunoblot for pRb in the eluent showed a significant stabilization of VHL interaction with pRb following proteasomal blockade (Fig. 1B), consistent with our mass spectrometry data. Similar results were observed in ccRCC-derived (RCC4) cells, wherein the exogenous expression of VHL-BirA promoted endogenous pRb biotinylation in a MG132-sensitive manner (Supplementary Fig. S1A). To determine if VHL and pRb interact, we exogenously expressed Flag-tagged pRb and HA-tagged VHL in HEK293A cells in the presence or absence of MG132. Immunoprecipitation of pRb revealed increased co-precipitation of VHL in the presence of MG132, verifying a proteasomal-sensitive interaction (Fig. 1C). To determine if VHL and pRb interact endogenously, we performed immunoprecipitation on MG132-treated HEK293A lysates using either an anti-VHL antibody or a non-targeting isotype control antibody. Immunoblot analysis showed co-precipitation of pRb with endogenous VHL (Fig. 1D). Similarly, a VHL-targeting antibody applied to lysates from MG132-treated ccRCC (786-O) cells stably reconstituted with HA-VHL, specifically co-precipitated pRb as compared to an isotype control (Supplementary Fig. S1B). Together, these findings indicate that pRb is a novel binding partner of VHL that may be targeted by the ubiquitin-proteasome pathway, similar to HIFα.

### VHL regulates pRb stability via the ubiquitin-proteasome pathway

**Fig. 2:**
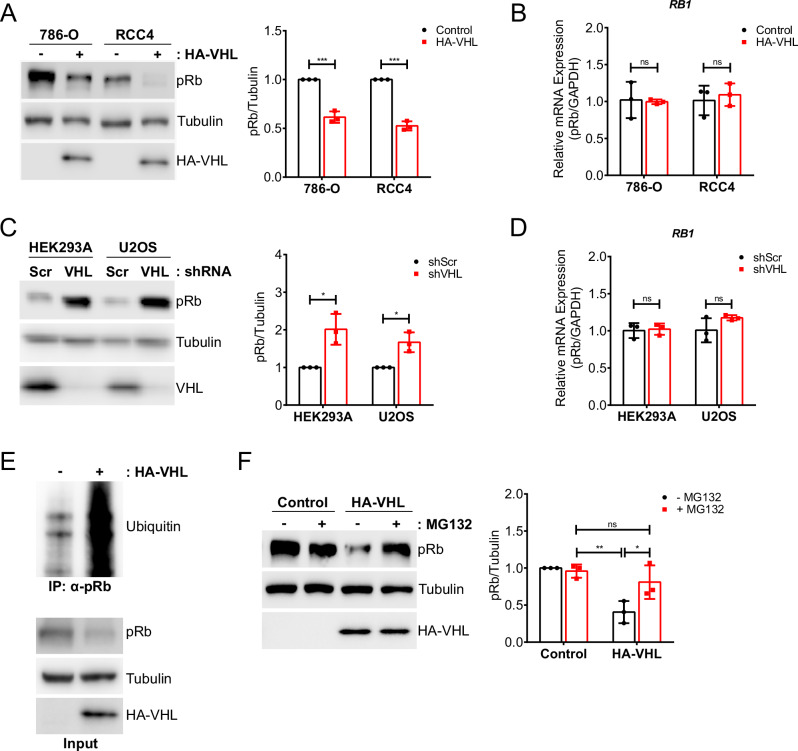
VHL promotes proteasomal degradation of pRb. **A** Immunoblot of lysates from 786-O and RCC4 cells stably transfected with either control vector (-) or hemagglutinin (HA)-tagged VHL. Quantification of pRb protein expression relative to tubulin is shown to the right. Statistical significance was calculated using unpaired t test and Holm-Sidak post-hoc test (*n* = 3). **B** pRb mRNA expression (relative to GAPDH) in 786-O and RCC4 cells stably transfected with either control vector or HA-tagged VHL. Fold changes in gene expression were calculated using the delta delta Ct method and normalized to the control condition. Statistical significance was calculated using unpaired t test and Holm-Sidak post-hoc test (*n* = 3). **C** Immunoblot of lysates from HEK293A and U2OS infected with lentivirus encoding either scrambled (Scr) or VHL-targeting shRNA. Quantification of pRb protein expression relative to tubulin is shown to the right. Statistical significance was calculated using unpaired t test and Holm-Sidak post-hoc test (*n* = 3). **D** pRb mRNA expression (relative to GAPDH) in HEK293A and U2OS cells infected with lentivirus encoding either scrambled (Scr) or VHL-targeting shRNA. Fold changes in gene expression were calculated using the delta delta Ct method and normalized to the control condition. Statistical significance was calculated using unpaired t test and Holm-Sidak post-hoc test (*n* = 3). **E** Immunoprecipitation under denaturing conditions of pRb from 786-O cells stably transfected with either control vector (-) or HA-tagged VHL and treated with 10 µM MG132 for 4 h. **F** Immunoblot of lysates from 786-O cells stably transfected with either control vector or HA-tagged VHL and treated as indicated with 10 µM MG132 or DMSO vehicle (-) for 4 h. Quantification of pRb protein expression relative to tubulin is shown to the right. Statistical significance was calculated using ordinary two-way ANOVA and Tukey’s post-hoc test (*n* = 3). **A**–**F** Data are represented as mean ± SD. *p < 0.05, **p < 0.01, ***p < 0.001. ‘ns’ denotes not significant.

To determine the effect of VHL on pRb expression, HA-tagged VHL was reconstituted into two VHL-deficient ccRCC cell lines, 786-O and RCC4. Cells were lysed at confluence to mitigate cell cycle variation in log-phase growth and any associated variability in pRb regulation by cell-cycle-dependent factors. Western blot analysis in both ccRCC lines showed that VHL re-expression leads to downregulation of pRb protein levels (Fig. 2A). This effect was determined to be post-transcriptional, as pRb mRNA levels were not affected by VHL reconstitution, as measured by quantitative real-time PCR (qRT-PCR) (Fig. 2B). Next, we sought to determine the impact of endogenous VHL knockdown in HEK293A and U2OS cells, which have functional VHL expression. In both cell lines, knockdown of VHL led to an increase in pRb protein expression (Fig. 2C). Consistent with our results in the ccRCC cell lines, VHL status did not affect pRb mRNA levels (Fig. 2D). Collectively, these results indicate that VHL regulates pRb at the post-transcriptional level.

Given that VHL interaction with pRb is stabilized by proteasomal inhibition, we next asked whether VHL could promote ubiquitination of pRb. To analyze VHL-mediated ubiquitination of pRb, we immunoprecipitated pRb under denaturing conditions from VHL-null and VHL-reconstituted 786-O cells treated with MG132. Immunoblot analysis showed significant pRb ubiquitination only in cells expressing VHL (Fig. 2E). Treatment of VHL-reconstituted 786-O cells with MG132 resulted in pRb protein accumulation comparable to VHL-null 786-O cells, whereas MG132 treatment of VHL-null cells did not significantly affect pRb protein levels (Fig. 2F). Together, these data demonstrate that VHL promotes the ubiquitination of pRb, thereby facilitating its proteasomal degradation.

### Transcriptional regulation by the VHL-pRb and VHL-HIF pathways are largely distinct

**Fig. 3:**
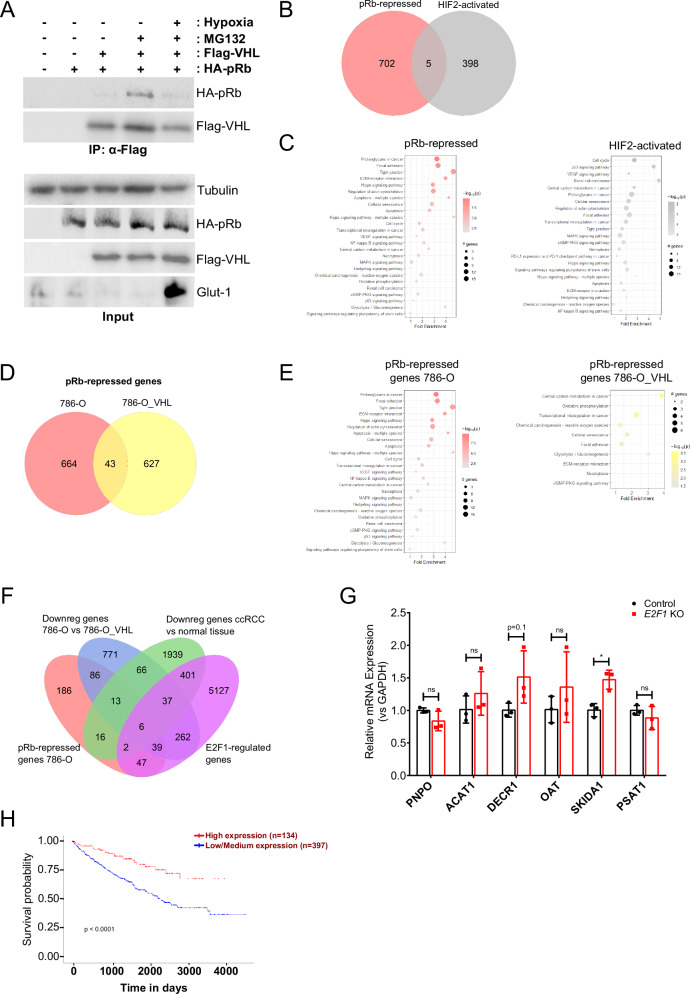
Transcriptional regulation by the VHL-pRb and VHL-HIF axes are distinct. **A** Immunoprecipitation of Flag-tagged VHL from HEK293A cells transfected with indicated plasmids and treated with 10 µM MG132 as indicated for 4 h. For hypoxia treatment, cells were placed in 0.5% O_2_ for 24 h. Glut-1 expression serves as positive control to indicate HIF upregulation under hypoxia. **B** Venn diagram showing overlap between genes upregulated by *RB1* KO in 786-O cells (red), and genes activated by HIF2α as obtained from the GSE149005 RNA-sequencing dataset (grey). Fold change > 1.5, adjusted p-value < 0.05. **C** Pathway enrichment analysis of pRb-repressed (red) and HIF-activated (grey) genes in 786-O cells using *pathfindR*. Enriched pathways were filtered based on biological relevance and visualized in a dot plot. **D** Venn diagram showing overlap between genes upregulated by *RB1* KO in VHL-null (red) and VHL-reconstituted (yellow) 786-O cells. Fold change > 1.5, adjusted p-value < 0.05. **E** Pathway enrichment analysis of pRb-repressed genes in VHL-null (red) and VHL-reconstituted (yellow) 786-O cells using *pathfindR*. Enriched pathways were filtered based on biological relevance and visualized in a dot plot. **F** Four-way Venn diagram showing overlap between the indicated gene sets, visualized using *ggvenn*. Fold change > 2, adjusted p-value < 0.05. E2F1-regulated transcripts were obtained from TRANSFAC database from ChIP-X enrichment analysis [86, 116]. Downregulated transcripts in ccRCC vs normal tissue were obtained from the International Cancer Genome Consortium (ICGC) data portal [12] and analyzed by DESeq2. Prognostic markers of ccRCC were obtained from the University of ALabama at Birmingham CANcer (UALCAN) [89] data analysis portal and based on Kaplan-Meier survival analysis [87]. **G** Analysis of mRNA expression (relative to GAPDH) of indicated genes in 786-O Cas9-expressing cells stably infected with virus encoding either control vector or *E2F1*-targeting guides. Fold changes in gene expression were calculated using the delta delta Ct method and normalized to the control condition. Statistical significance was calculated using unpaired t test and Holm-Sidak post-hoc test (*n* = 3). **H** Kaplan-Meier analysis showing the correlation between SKIDA1 expression in ccRCC and corresponding patient survival. **A**–**H** Data are represented as mean ± SD. *p < 0.05, **p < 0.01, ***p < 0.001. ‘ns’ denotes not significant.

The oxygen-dependent regulation of HIFα stability is important in the transcriptional reprogramming of cells under hypoxia [83]. pRb has also been described to repress hypoxia-regulated transcription [74]. However, the mechanism for oxygen-dependent pRb transcriptional regulation is not clearly understood. This led us to investigate whether VHL regulation of pRb was oxygen-sensitive. We analyzed the oxygen sensitivity of the pRb-VHL interaction in transfected cells. Immunoprecipitation of VHL showed that binding to pRb is inhibited by hypoxia (0.5% O_2_ for 24 hours), demonstrating that VHL interaction with pRb is oxygen-dependent (Fig. 3A). This suggests that VHL-mediated pRb regulation is involved in the cellular adaptation to hypoxia.

In response to oxygen limitation, cells upregulate pathways involved in angiogenesis, glucose metabolism and erythropoiesis to promote cellular adaptation to hypoxia [84]. pRb has also been described to regulate HIF-responsive genes in an oxygen-sensitive manner [49, 74]. We first asked whether pRb and HIF coordinate to transcriptionally regulate the same target genes. To identify pRb-regulated genes in ccRCC, we first created a monoclonal *RB1* knockout (KO) cell line from 786-O cells using CRISPR/Cas9. Next, we determined global mRNA expression in 786-O control and *RB1* KO cells using RNA-sequencing. We were interested in genes that were upregulated following *RB1* KO, as pRb is known to repress transcription. We found 707 genes with significantly higher expression in *RB1* KO cells compared to control cells (fold change >1.5, adjusted p-value (q) <0.05) (Fig. 3B, Supplementary Table S1). In contrast to pRb, HIFα is a transcriptional activator. HIF2a target genes were obtained from published datasets of RNA-sequencing analysis of 786-O control and *EPAS1* (HIF2α) KO in 786-O cells [85] (fold change >1.5, q < 0.05). Analysis of pRb-repressed and HIF2a-activated genes showed little commonality, indicating that pRb regulates a distinct set of genes from HIF2a (Fig. 3B). Despite having few common targets at the level of individual genes, KEGG pathway analysis of HIF and pRb-regulated gene sets showed enrichment of similar pathways such as focal adhesion, extracellular matrix (ECM)-receptor interaction, cell cycle, cellular senescence, and apoptosis (Fig. 3C). These results indicate that the transcriptional influence of VHL deletion in ccRCC is impacted by a combined dysfunction in HIF2a and pRb transcriptional regulation.

To determine pRb-regulated transcription in low vs high HIF2a-expressing cells, we created *RB1* knockouts in both VHL-reconstituted and VHL-null 786-O cells (Supplementary Figure S2). Following RNA-sequencing, we analyzed transcripts that were significantly upregulated in *RB1* KO cells compared to corresponding controls, to identify pRb-repressed genes. We identified 670 genes that were significantly repressed by pRb in VHL-reconstituted cells (Supplementary Table S2), compared to the 707 genes repressed in VHL-null 786-O cells as described above (fold change > 1.5, q < 0.05). Interestingly, comparison of both gene sets showed roughly 6% overlap, suggesting that pRb transcriptionally regulates a distinct set of genes based on HIF expression (Fig. 3D). However, KEGG pathway analysis of pRb-repressed transcripts in VHL-null and VHL-reconstituted cell lines showed enrichment of similar pathways such as focal adhesion, ECM-receptor interaction, necroptosis, oxidative phosphorylation, glycolysis, and cellular senescence (Fig. 3E). These tumor-associated pathways raise the possibility of the involvement of pRb in ccRCC oncogenesis.

We next sought to identify which transcriptional targets of pRb may contribute to ccRCC development. To narrow down the list of potential candidates, a stricter selection criteria of fold change >2 and q < 0.05 was applied. We omitted the pRb-repressed genes in cells with low HIF and VHL reconstitution, which is not a relevant state in most ccRCC, leaving 395 genes (Fig. 3F, Supplementary Tables S1 and S2). We then removed genes whose expression were not sensitive to VHL status (251 genes removed, Supplementary Table S3), leaving 144 genes. Among these VHL and pRb differentially expressed genes, we removed any that were not regulated by E2F-1 (obtained from ChIP-X Enrichment Analysis datasets [86]), as pRb has been described to repress apoptosis largely through E2F1 [54], leaving 45 genes. Finally, we asked which of these genes were repressed in ccRCC tumors versus normal tissue (Supplementary Table S4 - data obtained from the International Cancer Genome Consortium (ICGC)), which resulted in 6 genes (PNPO, ACAT1, DECR1, OAT, SKIDA1 and PSAT1) that fulfilled the following criteria: (1) repressed by pRb in 786-O cells (2) downregulated in VHL-deficient vs VHL-reconstituted 786-O cells (3) downregulated in ccRCC tumor vs normal tissue, and (4) regulated by E2F1 (Fig. 3F).

Among the selection criteria described above to identify physiologically relevant transcriptional targets of the VHL-pRb pathway, we have not demonstrated a requirement for E2F1 in our cell lines. To determine if the 6 narrowed down genes were regulated by E2F1 in ccRCC, we knocked out E2F1 from 786-O cells using CRISPR-Cas9 (Supplementary Fig. S3) and examined the effect on mRNA expression for each gene. qRT-PCR analysis demonstrated that SKI/DACH domain containing protein 1 (SKIDA1) is transcriptionally repressed in ccRCC cells in an E2F1-dependent manner (Fig. 3G). DECR1 and OAT mRNA levels were also increased upon E2F1 KO, but did not reach statistical significance. We next confirmed that SKIDA1 expression was upregulated by *RB1* deletion via qRT-PCR and immunoblot analyses of lysates from 786-O control and *RB1* KO cells (Supplementary Fig. S4). These results suggest that SKIDA1 may be a downstream target of the VHL-pRb pathway that is functionally repressed in ccRCC patients. Kaplan-Meier [87] survival analysis of ccRCC patients revealed a positive correlation between SKIDA1 expression in tumors and patient survival (Fig. 3H). These findings highlight SKIDA1 as a putative pRb target with prognostic significance in ccRCC, and whose repression upon VHL deletion may contribute to tumorigenesis.

### pRb inhibits apoptosis in ccRCC cells

**Fig. 4:**
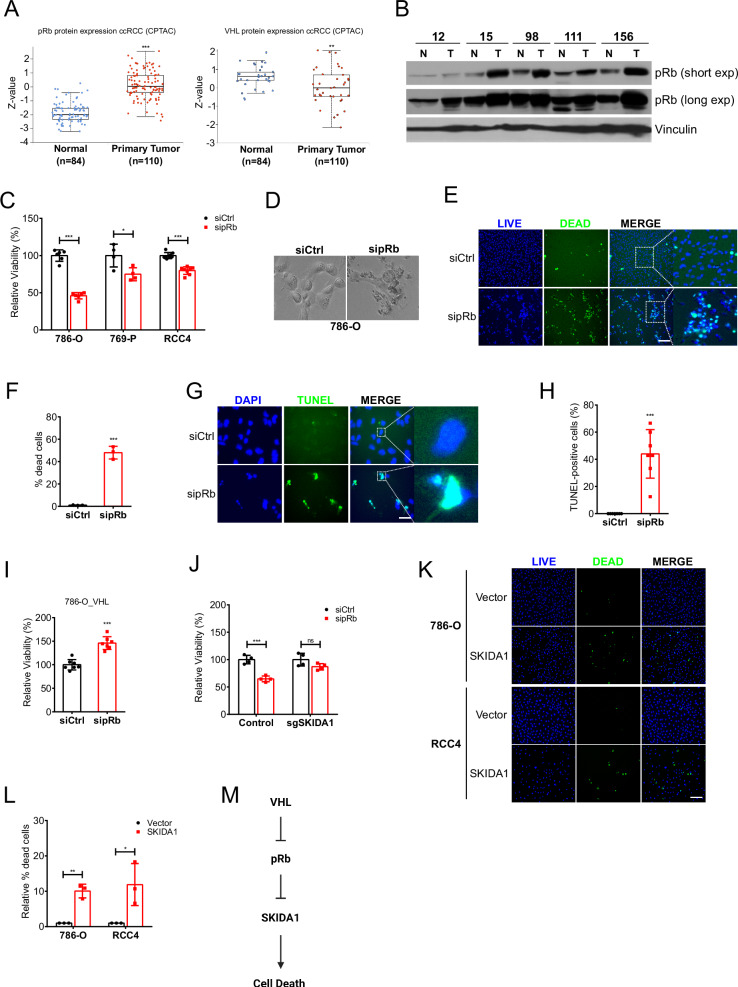
pRb suppresses apoptosis in ccRCC cells. **A** Analysis of pRb and VHL protein expression in ccRCC primary tumors and normal tissue. Patient data were obtained from Clinical Proteomic Tumor Analysis Consortium (CPTAC) dataset via the UALCAN data analysis portal. Z-values represent standard deviations from the median across normal and tumor samples. Log2 spectral count ratio values from CPTAC were first normalized within each sample profile, then normalized across samples. **B** Immunoblot of lysates from ccRCC primary tumor (T) and patient-matched normal tissue (N). **C** Analysis of cell viability (relative to control) in indicated ccRCC cell lines transfected with either non-targeting control siRNA (siCtrl) or pRb-targeting siRNA (sipRb), measured via a sulforhodamine B (SRB) assay. Statistical significance was calculated using unpaired t test and Holm-Sidak post-hoc test (*n* > 3). **D** Representative phase-contrast images of 786-O cells transfected with either non-targeting control siRNA (siCtrl) or pRb-targeting siRNA (sipRb). **E** Representative fluorescence images showing 786-O cells transfected with either non-targeting control siRNA (siCtrl) or pRb-targeting siRNA (sipRb), and stained using the ReadyProbes® Cell Viability Imaging Kit. The blue stain represents the total cells, while the green stain represents dead cells. Scale bar = 250 µm. **F** Quantification of percentage of dead cells relative to total number of cells in (E). Statistical significance was calculated using unpaired two-tailed t test (*n* = 3). **G** Representative images showing TUNEL staining of 786-O cells transfected with either non-targeting control siRNA (siCtrl) or pRb targeting siRNA (sipRb). Scale bar = 250 µm. **H** Quantification of percentage of TUNEL-positive cells relative to total number of cells in (G). Statistical significance was calculated using unpaired two-tailed t test (*n* = 7). **I** Analysis of cell viability (relative to control) in VHL-expressing 786-O cells transfected with either non-targeting control siRNA (siCtrl) or pRb targeting siRNA (sipRb), measured via a sulforhodamine B (SRB) assay. Statistical significance was calculated using unpaired two-tailed t test (*n* = 7). **J** Analysis of cell viability in 786-O Cas9-expressing cells transduced with either empty vector (control) or sgSKIDA1-containing vector, and transfected with either non-targeting control siRNA (siCtrl) or pRb targeting siRNA (sipRb). Cell viability was measured via a sulforhodamine B (SRB) assay and normalized to the siCtrl condition. Statistical significance was calculated using unpaired t test and Holm-Sidak post-hoc test (*n* = 4). **K** Representative fluorescence images of 786-O and RCC4 cells transduced with lentivirus containing either empty (control) plasmid (Vector) or SKIDA1 cDNA-encoding plasmid. Cells were stained using the ReadyProbes® Cell Viability Imaging Kit. The blue stain represents total cells, while the green stain represents dead cells. Scale bar = 250 µm. **L** Quantification (relative to control) of percentage of dead cells relative to total number of cells in (K). Statistical significance was calculated using unpaired t test and Holm-Sidak post-hoc test (*n* = 3). **M** Schematic illustration showing the VHL-pRb-SKIDA1 signalling axis and the effect on cell death. **A**–**M** Data are represented as mean ± SD. *p < 0.05, **p < 0.01, ***p < 0.001. ‘ns’ denotes not significant.

We have demonstrated that VHL promotes the degradation of pRb in a proteasomal sensitive manner. Therefore, pRb may be hyperstabilized in VHL-deficient tumors. Mass-spectrometry-based proteomic data obtained from the National Cancer Institute’s Clinical Proteomic Tumor Analysis Consortium (CPTAC) [88, 89] confirmed that pRb protein abundance is higher in ccRCC primary tumors (*n* = 110) compared to normal tissue (*n* = 84) (Fig. 4A), with high pRb levels linked to reduced progression-free survival in ccRCC patients, indicating some prognostic value [90]. As expected, analysis of the same samples showed VHL protein downregulation in ccRCC compared to normal tissue (Fig. 4A). Consistent with CPTAC data, immunoblot analysis showed that pRb was upregulated in the majority of ccRCC tumors compared to patient-matched normal tissue (Fig. 4B). These patient-derived data are consistent with our in vitro work in ccRCC cells, which showed a dramatic increase of pRb levels in cells lacking functional VHL (Fig. 2A).

To determine the effect of pRb hyperstabilization on the viability of ccRCC cells, we depleted pRb from several VHL-deficient ccRCC cell lines using pRb-targeting siRNAs (Supplementary Fig. S5) and analyzed cell viability using a sulforhodamine B (SRB) colorimetric assay. We observed a significant decrease in cell viability following pRb depletion in all ccRCC cell lines (Fig. 4C). By microscopy, we observed morphological changes associated with cell death including cell shrinkage, rounding, and increased cellular debris in pRb-depleted 786-O cells (Fig. 4D). Live/dead cell staining of 786-O cells confirmed an increase in the proportion of dead cells upon siRNA-mediated knockdown of pRb (Fig. 4E, F). Several reports have shown that pRb can repress apoptosis, while others have reported a repression of non-apoptotic cell death [54, 72, 74, 91–95]. To determine if cell death induced by pRb depletion was apoptotic, we performed terminal deoxynucleotidyl transferase dUTP nick end labeling (TUNEL) staining on 786-O control and pRb knockdown cells. We observed a significant increase in the proportion of TUNEL-positive (or apoptotic) cells following pRb knockdown, suggesting that pRb inhibits apoptosis in ccRCC cells (Fig. 4G, H). Of note, pRb loss does not contribute to cellular senescence, a pathway that was enriched in our analysis of pRb transcriptional targets (Fig. 3E, Supplementary Fig. S6). Interestingly, pRb knockdown in VHL-reconstituted 786-O cells did not lead to a similar decrease in cell viability, in stark contrast to the effect of pRb depletion in VHL-null cells (Fig. 4I, Supplementary Fig. S7). These findings suggest that the anti-apoptotic function of pRb in ccRCC cells may be dependent on VHL or HIF expression, which is consistent with the differential transcriptional regulation by pRb in the presence or absence of VHL (Fig. 3D) and the synthetic lethality observed between *Vhl* and *Rb1* [49].

We next asked whether the downstream VHL-pRb transcriptional target SKIDA1 contributes to the induction of cell death following pRb knockdown. Since SKIDA1 is upregulated upon knockout of *RB1*, we knocked out *SKIDA1* in 786-O cells and analyzed the effect of pRb depletion on cell viability as measured by an SRB assay. Knockout of *SKIDA1* rescued viability of 786-O cells depleted of pRb (Fig. 4J). Next, we tested if overexpression of SKIDA1 alone was sufficient to induce cell death. Two ccRCC cell lines were infected with lentivirus encoding either control vector or SKIDA1 expression vector (Supplementary Fig. S8). Live/dead cell staining showed a significant increase in the proportion of dead cells following SKIDA1 overexpression in both cell lines (Fig. 4K, L). Collectively, our results suggest that pRb suppresses cell death specifically in VHL-deficient renal cells, likely via regulation of downstream transcriptional targets such as SKIDA1 (Fig. 4M).

### pRb depletion inhibits ccRCC tumorigenesis

**Fig. 5:**
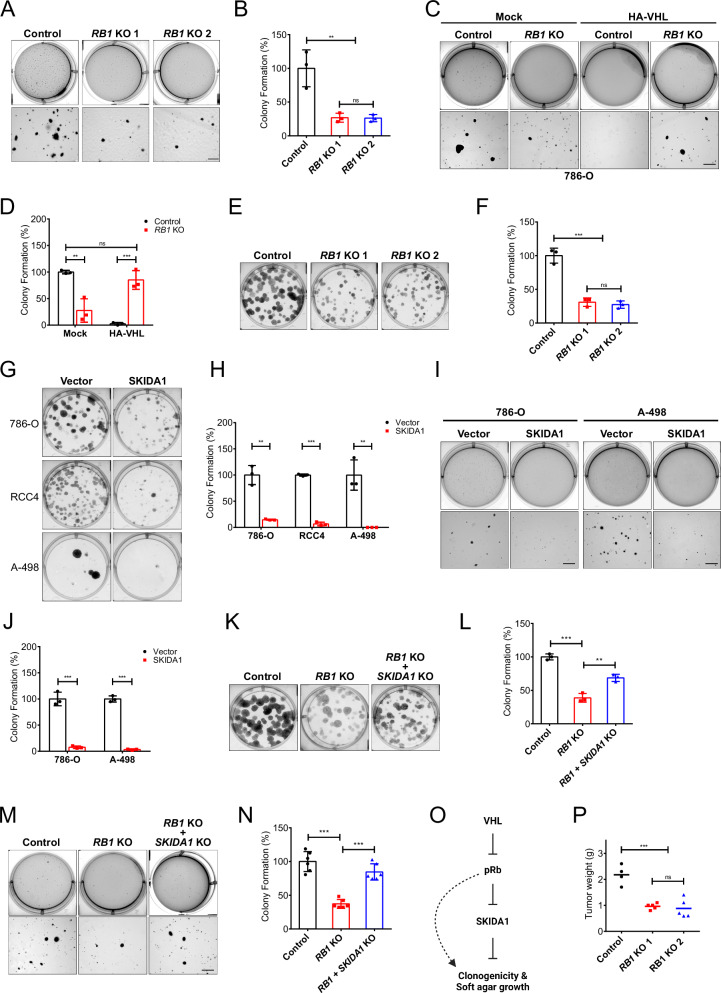
pRb hyperstabilization promotes ccRCC oncogenesis. **A** Representative images showing colony formation of 786-O control and *RB1* KO cells in soft agar. Scale bar = 500 µm. **B** Quantification (relative to control) of the number of colonies formed in **A**. Colonies were manually counted under a light microscope. Statistical significance was calculated using ordinary one-way ANOVA and Tukey’s post-hoc test (*n* = 3). **C** Representative images showing colony formation in soft agar of 786-O control and *RB1* KO cells stably transfected with either control vector (Mock) or HA-tagged VHL. Scale bar = 500 µm. **D** Quantification (relative to control) of the number of colonies formed in **C**. Colonies were manually counted under a light microscope. Statistical significance was calculated using ordinary two-way ANOVA and Tukey’s post-hoc test (*n* = 3). **E** Representative images showing clonogenic outgrowth of 786-O control and *RB1* KO cells. **F** Quantification (relative to control) of the number of colonies in **E**. Statistical significance was calculated using ordinary one-way ANOVA and Tukey’s post-hoc test (*n* = 3). **G** Representative images showing clonogenic outgrowth of 786-O, RCC4 and A-498 cells stably infected with lentivirus containing either control plasmid (Vector) or SKIDA1 cDNA containing plasmid. **H** Quantification (relative to control) of the number of colonies in (G). Statistical significance was calculated using unpaired t test and Holm-Sidak post-hoc test (*n* = 3). **I** Representative images showing colony formation in soft agar of 786-O and A-498 cells stably infected with lentivirus containing either empty (control) plasmid (Vector) or SKIDA1 cDNA-encoding plasmid. Scale bar = 500 µm. **J** Quantification (relative to control) of the number of colonies formed in (**I**). Colonies were manually counted under a light microscope. Statistical significance was calculated using unpaired t test and Holm-Sidak post-hoc test (*n* = 3). **K** Representative images showing clonogenic outgrowth of 786-O control, *RB1* KO and *RB1*/*SKIDA1* double KO cells. **L** Quantification (relative to control) of the number of colonies in **K**. Statistical significance was calculated using ordinary one-way ANOVA and Tukey’s post-hoc test (*n* = 3). **M** Representative images showing colony formation in soft agar of 786-O control, *RB1* KO and *RB1*/*SKIDA1* double KO cells. Scale bar = 500 µm. **N** Quantification (relative to control) of the number of colonies formed in **M**. Colonies were manually counted under a light microscope. Statistical significance was calculated using ordinary one-way ANOVA and Tukey’s post-hoc test (*n* = 6). **O** Schematic illustration showing the VHL-pRb-SKIDA1 signalling axis and the effect on clonogenicity and soft agar growth. The dotted line suggests alternate pathways. **P** Scatter dot plot showing quantification of weights of tumors formed by 786-O control and *RB1* KO cells injected subcutaneously into the flanks of immunodeficient *NOD-scid IL2Rg*^*null*^ mice, via a tumor xenograft assay. The line indicates the mean. Five injections were performed with control cells and ten injections with *RB1* KO cells (five for each clone). One control injection which failed to grow was considered an outlier and omitted during statistical analysis. Statistical significance was calculated using ordinary one-way ANOVA and Tukey’s post-hoc test (*n* > 3). **A**–**P** Data are represented as mean ± SD. *p < 0.05, **p < 0.01, ***p < 0.001. ‘ns’ denotes not significant.

pRb-mediated repression of cell death in ccRCC may be indicative of oncogenic contribution of hyperaccumulated pRb in ccRCC. Therefore, we sought to determine the effects of pRb accumulation on ccRCC cancer phenotype. We first analyzed anchorage-independent growth of ccRCC cells in soft agar, which is an important hallmark of cancer [96–98]. 786-O cells are known to grow in an anchorage-independent manner [41, 85], so in this background we created two independent *RB1* KO cell lines using CRISPR-Cas9 (Supplementary Fig. S9). Next, using 786-O control and *RB1* KO cells, we measured anchorage-independent growth in the soft agar 3D culture model. We found that knockout of *RB1* significantly inhibited anchorage-independent growth of 786-O cells (Fig. 5A, B). In contrast, knockout of *RB1* in VHL-reconstituted 786-O cells was sufficient to induce anchorage-independent growth (Fig. 5C, D). Interestingly, knockdown of pRb in 786-O cells to levels similar to VHL-reconstituted cells, was sufficient to inhibit anchorage-independent growth (Supplementary Fig. S10). These data are consistent with an oncogenic role for hyperaccumulated pRb specifically in VHL-deficient ccRCC.

**Fig. 6:**
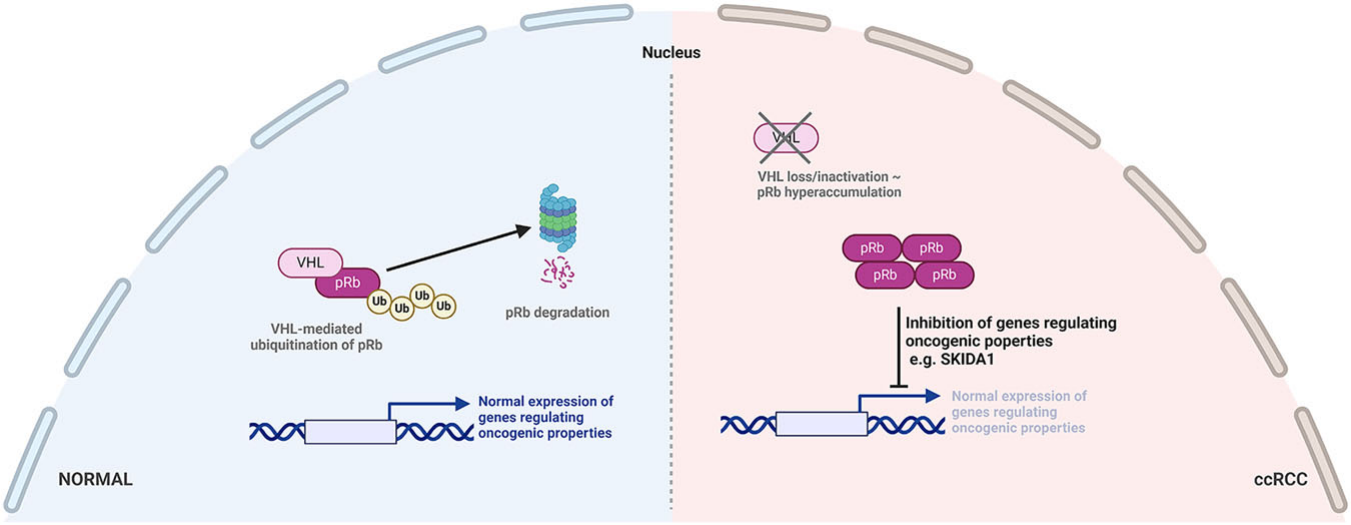
Schematic diagram illustrating the pRb regulation by VHL and effects on downstream targets. In normal kidney cells, VHL regulation of pRb promotes normal expression of genes regulating oncogenic properties. In ccRCC, VHL loss promotes pRb hyperaccumulation, which leads to the repression of genes regulating oncogenic properties.

We next assessed the clonogenic capacity of 786-O control and *RB1* KO cells by performing a clonogenic assay, a survival assay based on the ability of a single cell to grow into a colony. Consistent with our findings from the soft agar assay, we observed a significant decrease in clonogenic outgrowth following pRb depletion (Fig. 5E, F, Supplementary Fig. S11). Together, these results indicate that pRb is required to maintain the tumorigenic potential of ccRCC cells. We next looked downstream at the potential impact of SKIDA1 on ccRCC clonogenicity and anchorage-independent growth. We found that upregulation of the pRb target SKIDA1 in ccRCC cells led to a significant decrease in clonogenic outgrowth (Fig. 5G, H) and anchorage-independent growth in soft agar (Fig. 5I, J). Furthermore, depletion of SKIDA1 in 786-O *RB1* KO cells resulted in partial rescue of colony formation (Fig. 5K, L) and anchorage-independent growth in soft agar (Fig. 5M, N). These results further emphasize that SKIDA1 may be an important downstream tumor suppressor target of the VHL-pRb pathway in ccRCC (Fig. 5O). To examine the effect of *RB1* knockout on ccRCC tumor growth in vivo, we injected 786-O control and *RB1* KO cells subcutaneously into the flanks of immunodeficient *NOD-scid IL2Rg*^*null*^ (NSG) mice. All mice were euthanized when the first tumor reached endpoint. Tumors were then excised and weighed. One control injection which failed to grow was considered an outlier and omitted during statistical analysis. We found that on average, tumors formed by 786-O control cells were significantly larger compared to those formed by *RB1* KO cells (Fig. 5P). Furthermore, immunohistochemical staining of SKIDA1 in extracted tumors revealed increased SKIDA1 expression in *RB1* KO tumors compared to control tumors (Supplementary Fig. S12). To further validate SKIDA1 as a clinically relevant target in ccRCC, we performed immunoblot analysis of additional ccRCC tumor and patient-matched normal samples, genotyped to confirm VHL mutation in all tumor samples. As expected, pRb protein expression was upregulated in the majority of tumors (Supplementary Fig. S13). And whilst SKIDA1 expression varied significantly between tumors, overall, we observed downregulation of SKIDA1 protein levels in most tumors compared to their matched normal tissue, further underscoring the clinical significance of our findings. Collectively, our results indicate that pRb hyperstabilization promotes ccRCC tumorigenesis, likely through transcriptional remodelling including the identified target SKIDA1 (Fig. 6).

## Discussion

The incidence rate of ccRCC is 8.01 per 100,000 population in patients at least 40 years of age [99]. ccRCC is rarely diagnosed at early stages and tumors at advanced stages are extremely resistant to chemotherapy and radiation therapy [100]. While combination therapy with immune checkpoint inhibitors and anti-angiogenic drugs show improved antitumor efficacy, issues relating to systemic toxicity and drug resistance persist, necessitating research into novel therapeutically tractable pathways [39, 101]. Using an unbiased screen to discover novel targets of VHL, we identified pRb as a VHL substrate that is regulated by ubiquitination and subsequent proteasomal degradation. pRb depletion led to decreased clonogenicity and anchorage-independent growth of ccRCC cells. Loss of pRb also led to reduced ccRCC tumorigenesis in vivo using mouse xenograft models, highlighting the tumorigenic activity of pRb in ccRCC. Transcriptomic analysis identified SKIDA1 as an important downstream target of the VHL-pRb pathway that is repressed in ccRCC. Overexpression of SKIDA1 significantly decreased ccRCC clonogenicity and anchorage-independent growth. Further characterization of the mechanisms of SKIDA1’s tumor suppressive function in ccRCC and expanded characterization of other VHL-pRb regulated transcriptional targets in ccRCC is needed to appreciate the scope and mechanism of pRb dysregulation in ccRCC. In this paper, the characterization of pRb hyperstabilization in ccRCC provides a sound rationale for molecular targeting of pRb and its disease-related targets such as SKIDA1.

Although pRb is best known as a tumor suppressor, in this study, we describe a context-specific potential for this protein to act as an oncogene when dysregulated by VHL loss. *RB1* is not a common target for inactivating mutations in ccRCC, so in theory, pRb should be able suppress cell cycle. However, this function is mediated by the unphosphorylated form of pRb and in ccRCC, pRb is inactivated by hyperphosphorylation [55, 102, 103]. The inactivation of the tumor suppressive function of wild type pRb in ccRCC is likely due to the reported cyclin D1 overexpression, fueled by HIF activation, as well as the inactivation of CDK inhibitors, leading to phosphorylation-based inactivation of cell cycle control [62, 104]. Hyperphosphorylated pRb likely promotes tumorigenesis through mechanisms independent of cell cycle. Several studies have described cell-cycle-independent functions of pRb. For example, studies on homozygous *Rb1* deletion in mouse embryos showed significant cell death in several tissues including the eye lens, nervous system, and skeletal muscle [72, 92, 105]. These *Rb1* mutant embryos also fail to reach term. Our findings also highlight a context-dependent role for pRb in ccRCC whereby pRb functions as an oncogene in the absence of VHL, and as a tumor suppressor in the presence of VHL. As such, targeting of pRb in ccRCC may be restricted to tumors that lack functional VHL expression. This is conceptually consistent with a recent study showing dual inhibition of CDK 4 and 6 is synthetically lethal with VHL loss, which acted in a pRb-dependent manner [50]. This synthetic lethality was observed in a conserved manner in flies and mammals. Additionally, dual knock out of *Vhl* and *Rb1* in the mouse retina resulted in synthetic lethality in rods and cone cells, further underscoring the epistatic links between VHL and pRb [49]. Both above studies highlight a link between VHL and pRb in regulating cell viability. In this study, we identified that VHL loss alters pRb stability and transcriptional regulation. These transcriptional targets of pRb, including SKIDA1, contribute to apoptosis in ccRCC cells. Collectively, our study and those listed above supports a rationale for targeting pRb and its downstream transcriptional targets in ccRCC.

The function of SKIDA1 is relatively understudied. It was found to be differentially expressed in the regulatory T cells of Autoimmune polyendocrine syndrome type I (APS-1) patients compared to healthy patients [106]. SKIDA1 was also described as the best predictor of *KMT2A* (Mixed-lineage-leukemia) gene rearrangements [107]. However, its role in tumor development has not been defined. The understudied nature of SKIDA1 and its prognostic value in ccRCC patient survival made it a particularly interesting target to follow up on. Here we describe a role for SKIDA1 in inhibiting ccRCC tumorigenesis by promoting cell death. Mechanistically, SKIDA1 repression in ccRCC may be mediated by the pRb-E2F1 repressive complex as depletion of either pRb or E2F1 rescues SKIDA1 expression in ccRCC cells. This supports previous findings that pRb can regulate E2F1-driven transcription of apoptotic genes [54, 108, 109]. While our experiments point towards transcriptional regulation of SKIDA1 by the pRb-E2F1 complex, chromatin immunoprecipitation (ChIP) experiments will be needed to validate direct regulation of SKIDA1 by these transcription factors. In this study, we examine VHL-pRb regulation in the context of disease. We also noted the oxygen-sensitive interaction of pRb by VHL, suggesting that this regulation may play an important role in the cellular adaptation to hypoxia. Further characterization of the VHL-pRb signaling pathway in normal cells may promote our understanding of the cellular hypoxia response and the interplay with disease development.

Although not clinically approved for the treatment of ccRCC, CDK4/6 inhibitors have shown promise when combined with other therapeutics in preclinical mouse models and clinical trials [50, 110, 111]. By inhibiting the phosphorylation of pRb, CDK inhibitors can reverse the hyperphosphorylated pRb phenotype in ccRCC, thereby promoting cell cycle arrest. Studies using the CDK4/6 inhibitor Palbociclib and the HIF-2α inhibitor PT2399 have demonstrated synergy in suppressing the viability of HIF-2α–dependent ccRCC cell lines [50]. Based on our findings, the coupling of CDK inhibitors and E2F1 activators may also contribute synergistic activity in VHL-deficient ccRCC. E2F-1 activators have previously been shown to promote checkpoint-mediated apoptosis in tumorigenic cells, but haven’t been tested in conjunction with CDK4/6 inhibitors [112, 113]. Alternatively, SKIDA1 activators, though not yet identified, represent another promising avenue for inducing tumor cell death. For increased efficacy and to minimize resistance, combining drugs with distinct mechanisms of action is likely to provide the best outcome in the context of ccRCC. As a result, CDK4/6 inhibitors, E2F1 activators, and potential SKIDA1 activators should therefore be interrogated in conjunction with established frontline therapies such as immune checkpoint inhibitors, tyrosine kinase inhibitors, and mTOR inhibitors, to determine new and possibly superior treatment modalities. Overall, our findings highlight pRb as a novel target for VHL-mediated degradation and the VHL-pRb-SKIDA1 pathway as a potential therapeutic target in ccRCC treatment.

## Materials and methods

### Cell culture and reagents

All cell lines used in cell culture were obtained from the American Type Culture Collection (ATCC). 786-O, 769-P and RCC4 cells were grown in RPMI 1640 medium (350-000-CL, Wisent) supplemented with 10% (vol/vol) fetal bovine serum (FBS). A-498, 293 A and U2OS cells were grown in Dulbelcco’s modified Eagle’s medium (319-015-CL, Wisent) supplemented with 10% (vol/vol) FBS. All cells were maintained in a humidified incubator at 37 °C, 5% CO_2_, and 21% O_2_ (normoxia). Assays were performed on arrested cultures grown to confluence in order to control for pRb cell cycle function. Moreover, VHL-mediated pRb degradation was more prominent in confluent cells, suggesting a confluence-dependent mechanism for VHL-pRb regulation. For hypoxic treatments, cells were placed in a hypoxia chamber at 37 °C, 5% CO_2_, and 0.5% O_2_ for 24 hours. MG132 (Peptides International) was used at 10 µM. Cell lines were routinely tested for mycoplasma contamination.

### Western blot and antibodies

Whole cell lysates were prepared by lysing cells in 1x Laemmli buffer. After boiling at 95 °C for 10 minutes, samples were separated by SDS-PAGE and transferred on to polyvinylidene fluoride (PVDF) membranes. After blocking, membranes were incubated in primary antibody diluted in 5% bovine serum albumin (BSA) in TBST. Membranes were washed with TBST and incubated with the appropriate horseradish peroxidase (HRP)-conjugated secondary antibody diluted in 2% non-fat milk in TBST. After washing, blots were developed with the chemiluminescence method and imaged using the Bio-Rad ChemiDoc imaging system.

Antibodies against pRb (9313), HA (2999), VHL (68547), and Ubiquitin (3936) were from Cell Signaling Technology. Antibodies against Vinculin (V9131), β-actin (A5441), α-tubulin (T6199) and Flag (A8592) were from Sigma Aldrich. Anti-HIF2α (NB100-122) antibody was from Novus Biologicals. Glut-1 (115730) antibody was from Abcam. Anti-SKIDA1 (ARP69749_P050) antibody was from Aviva systems biology. Peroxidase-conjugated sheep anti-mouse (NB1206808) and donkey anti-rabbit (NB7185) antibodies were purchased from Novus Biologicals. Antibodies used for co-immunoprecipitation include mouse anti-HA (homemade), anti-Flag M2 (A2220, Sigma), mouse anti-pRb (554136, BD Biosciences), and mouse anti-VHL (556347, BD Biosciences). High-Capacity Streptavidin Agarose beads (20359, Thermo Fisher Scientific) were used for biotin affinity purification.

### Quantitative real-time polymerase chain reaction (qRT-PCR)

Total RNA was extracted from cells using the PureLink RNA Mini Kit (12183018 A, Invitrogen) according to the provided protocol. On-column genomic DNA digestion was performed using the PureLink DNase Set (12185010, Invitrogen). First strand cDNA synthesis was performed with the iScript cDNA Synthesis Kit (#1708891, Biorad) according to provided protocol. Real-time quantititative PCR (qRT-PCR) detection was performed using the Luna® Universal qPCR Master Mix (M3003, New England Biolabs) and the CFX96 Real-Time PCR System. 10 ng of cDNA was used as input for qRT-PCR reactions. Real-time PCR was performed in triplicate and relative gene expression was calculated using the 2^-ΔΔCt^ method [114] following normalization of Ct values to a GADPH control. Gene-specific primers were designed using the IDT PrimerQuest tool.

Real-time PCR primers (forward -F and reverse -R) used are as follows: RB1 (**F:** AATCAGATGGTATGTAACAGCGA; **R:** TGAAATTTGGACTCTCCTGGG), SKIDA1 (**F:** GTCCTGAATTTGCTACGGATTTG; **R:** CAGGGATTCTCGGCTTTAGTT), ACAT1 (**F:** TGAACAGGACGCTTATGCTATT; **R:** CCACTACATCTGGTTGACCTTT), DECR1 (**F:** GTGATTCAACCAGGGCCTATAA; **R:** CAGGGAATTCTGCCAATCATTTC), PSAT1 (**F:** GCTTGGTTCTGGAGTGGATTA; **R:** CTCCACTGGACAAACGTAGAA), PNPO (**F:** TGATCGGGAGTATCTGAGAAAGA; **R:** ACCTGAGGGTACAGGACATAG), OAT (**F:** GTAGATGGCTGGCTGTTGATTA; **R:** CACTGCAGACACAGGGTATAAG), E2F1 (**F:** CCTGCAGAGCAGATGGTTAT; **R:** GCTCTTAAGGGAGATCTGAAAGT), GAPDH (**F:** GGTGTGAACCATGAGAAGTATGA; **R:** GAGTCCTTCCACGATACCAAAG).

### Virus production and transduction

HEK293T cells were used for lentivirus production. The packaging plasmids psPAX2 (#12260) and pMD2.G (#12259) were purchased from Addgene. Viruses were harvested at 48 h and 72 h post-transfection, filtered through 0.45μm PVDF filter and immediately used to infect target cells. The virus-containing media was supplemented with 8 µg/ml polybrene to improve transduction efficiency. Transduced cells were then selected for using the appropriate antibiotic. Stable cell lines were created by selecting for viral integration using 1 µg/ml puromycin or 0.5 mg/ml G418, as appropriate.

### Transfection and co-immunoprecipitation assays

293 A/T cells were transfected using polyethylenimine (PEI) (23966-1, Polyscience) reagent 48 hours prior to lysis. Transfection of 786-O cells was achieved by electroporation using the Amaxa Nucleofector 2b device (Lonza) and manufacturer recommended conditions. siRNAs were transfected using Lipofectamine RNAiMAX transfection reagent (13778030, Thermo Fisher Scientific).

For immunoprecipitation, cells were harvested in mild lysis buffer (10 mM Tris pH 7.5, 100 mM NaCl, 10 mM EDTA, 50 mM NaF, 1% NP-40) supplemented with complete protease inhibitor. Lysates were clarified by centrifugation, and then incubated with the appropriate antibody and beads for 1 hour. Bound complexes were washed with mild lysis buffer and eluted by boiling in 1x Laemmli buffer at 95 °C for 10 minutes. Bound proteins were then resolved using SDS-PAGE followed by western blot analysis.

For immunoprecipitation under denaturing conditions, cells were lysed in mild lysis buffer and clarified by centrifugation. Sodium dodecyl sulphate (SDS) was added to cell lysate to a final concentration of 1%, after which the lysate was boiled at 95 °C for 5 minutes to denature proteins and disrupt protein-protein interactions. The cooled lysate was then diluted with mild lysis buffer to a final concentration of 0.1% SDS prior to immunoprecipitation. Mouse anti-pRb antibody (554136, BD Biosciences) was used to pull down denatured pRb.

### Proximity labeling assay

24 hours post-transfection of mammalian cells with plasmid encoding VHL-Flag-BirA, 10 µM MG132 or DMSO vehicle was added for 4 hours. 50 µM biotin was then added onto appropriate cells for an additional hour. Cells were lysed with high-salt RIPA buffer and snap-frozen in liquid nitrogen. Lysates were thawed rapidly in a 37 °C water bath and clarified by sonication followed by centrifugation. Salt concentration of lysates was halved using no-salt RIPA buffer. Lysates were then pre-cleared using protein G agarose beads (16-266, Sigma Aldrich) at 4^°^C for 10 minutes. The pre-cleared lysates were incubated with streptavidin-agarose beads (20359, Thermo Fisher Scientific) at 4^°^C for 4 hours to affinity purify biotinylated proteins. Biotinylated proteins were then eluted off the beads using 30 mM biotin (B4639, Sigma Aldrich). The samples were resolved on a NuPAGE 10% Bis-Tris gel (NP0315BOX, Thermo Fisher Scientific) and visualized using the SimplyBlue SafeStain (465034, Thermo Fisher Scientific). Bands were then excised and destained, followed by in-gel trypsin digestion.

An aliquot of each tryptic digest was analyzed by liquid chromatography–tandem mass spectrometry on an Orbitrap Fusion Lumos system (Thermo Scientific) coupled to a Dionex UltiMate 3000 RSLC nano HPL. The raw files were searched against the Human UniProt Database using MaxQuant software v1.2.4.0 and the following criteria used: peptide tolerance = 10 ppm, trypsin as the enzyme (2 missed cleavages allowed), and carboxyamidomethylation of cysteine as a fixed modification. Variable modifications are oxidation of methionine and N-terminal acetylation. The peptide and protein FDR was 0.01.

### Live/dead cell staining

Live/dead cell staining was performed using the ReadyProbes® Cell Viability Imaging Kit (Blue/Green) (R37609, Thermo Fisher Scientific). 2 drops of each reagent were added to 1 ml of cell culture media. Cells were then incubated with reagent-containing media for 15 minutes at room temperature. The NucBlue® Live reagent stains all nuclei whereas the NucGreen® Dead reagent stains only the nuclei of cells with compromised plasma membrane integrity. Images were obtained using the EVOS M5000 microscope at 10x objective. The Fiji software was used for image analysis and adjustment of image parameters.

### TUNEL assay

Adherent cells were rinsed twice with PBS and fixed with 4% paraformaldehyde (PFA) for 1 hour at room temperature. Cells were rinsed with PBS, then permeabilized with 0.1% Triton X-100 in 0.1% sodium citrate for 2 minutes on ice. Cells were rinsed twice with PBS. 50 µl TUNEL reaction mixture (11684795910, Sigma Aldrich) was added to cell monolayer and incubated for 60 minutes at 37 °C in a humidified atmosphere in the dark. Cells were then rinsed 3 times with PBS and counterstained with 1 µg/ml 4′,6-diamidino-2-phenylindole (DAPI) reagent (D9542, Sigma Aldrich). For negative control, cells were incubated in 50 µl/well Label solution (without terminal transferase) instead of TUNEL reaction mixture. For positive control, cells were incubated with DNase I (3 U/ml in 50 mM Tris-HCl pH 7.5, 10 mM MgCl_2_, 1 mg/ml BSA) for 10 minutes at room temperature to induce DNA strand breaks prior to labeling. Images were obtained using the EVOS M5000 microscope at 10x objective. The Fiji software was used for image analysis and adjustment of image parameters.

### Sulforhodamine B (SRB) assay

Cells were fixed in media containing 10% trichloroacetic acid (TCA) (TB0968, Bio Basic) at 4 ^°^C for 2 hours. After washing off fixation solution, cells were stained with 0.04% (wt/vol) Sulforhodamine B sodium salt (HY-D0974, MedChemExpress) for 30 minutes. Cells were rinsed with 1% (vol/vol) acetic acid to remove unbound dye. 150 µl of 10 mM Tris base solution (pH 10.5) was then added to solubilize the protein-bound dye. Dye absorbance (570 nm) and background absorbance (650 nm) were obtained using a microplate reader. Final absorbance reading was obtained by subtracting background absorbance from dye absorbance.

### Soft-agar colony formation assay

The soft agar colony formation assay was done on 6-well plates. The bottom layers consisted of 1.5 ml of 1% low melting point agarose (16520050, Thermo Fisher Scientific) in 1X RPMI media supplemented with FBS. The upper layers consisted of 7500 cells embedded in 1.5 ml of 0.5% agarose in 1X RPMI media supplemented with FBS. Both layers were allowed to solidify for 5 minutes at 4°C, then placed in a 37 °C incubator for the remainder of the assay. Every 3 days, 200 µL of complete media were added onto the semi-solid media to prevent desiccation. After 5 weeks, colonies were stained with 200 µg/ml iodonitrotetrazolium chloride (IB0280, Bio Basic) solution. Colonies were counted under a light microscope. Colonies greater than 100 µm in diameter were quantified. Images were obtained using the Bio-Rad ChemiDoc imaging system and the EVOS M5000 microscope.

### Clonogenic assay

150 cells were seeded on a 6-well plate and cultured for 10 days. Cell culture media was replaced every 3-4 days. Cells were rinsed with PBS, fixed with 3 vol methanol + 1 vol acetic acid for 5 minutes and stained with 0.5% (wt/vol) crystal violet in methanol for 15 minutes. Stained cells were rinsed with tap water until background was clear. Images of colonies were obtained using the Bio-Rad ChemiDoc imaging system.

### β-galactosidase assay

Cells were stained using a senescence-associated β-galactosidase staining kit (602010, Cayman Chemical), according to manufacturer’s protocol. Briefly, cells were washed twice with PBS and fixed for 15 minutes at room temperature. Cells were then washed twice with PBS, followed by incubation in cell staining solution (pH 6) at 37 °C overnight. Brightfield images were then obtained using the EVOS M5000 microscope.

### RNA sequencing and data analysis

Total RNA was extracted using Trizol reagent (15596026, Thermo Fisher Scientific). RNA was initially quality controlled by Qubit and Tapestation. Libraries were prepared using the Truseq kit from Illumina using the manufacturer’s protocol (https://www.illumina.com/products/by-type/sequencing-kits/library-prep-kits/truseq-stranded-mrna.html). Quantity and quality of libraries were assessed by qPCR and LabChip, respectively. All libraries were sequenced on 1 lane of NovaSeq S1 (PE100), to generate 50 million reads per condition.

Raw sequencing data was processed using GenPipes [115] with standard settings, which aligns reads to GRCh37 (hg19) with *STAR* 2-passes mode and counted to gene features using ‘htseq-count’ function from *HTSeq*. The raw count data was normalized and analyzed by *DESeq2*. The differentially expressed genes (DEG) (adjusted p-value < 0.05, fold change > 1.5 or > 2.0) were compared to HIF2α-activated genes (downloaded from GSE149005 and re-analyzed by *limma* for significant DEGs with adjusted p-value < 0.05), downregulated genes in ccRCC versus normal kidney tissues (downloaded from the International Cancer Genome Consortium (ICGC) and analyzed by DESeq2), and E2F1-regulated genes (downloaded from TRANSFAC database from ChIP-X enrichment analysis [86, 116]). The comparisons between the DEGs in each group were visualized using *ggvenn*, and the pathway enrichment analysis was done using *pathfindR*. The enriched pathways were filtered based on biological relevance and visualized in a dot plot.

### Patient samples

Patient samples were provided by McGill/McGill University Health Centre (MUHC) RCC biobank, and had been collected after written consent was obtained from patients and the study has been approved by MUHC IRB. Proteins were extracted from tissue samples using RIPA buffer and mechanical lysis in a dounce homogenizer. Lysates were then clarified by centrifugation and 4x Laemmli Sample Buffer added to 1x final concentration.

### Tumor xenograft assay

For tumor xenografts, four male and four female *NOD-scid IL2Rg*^*null*^ (strain JAX:005557, Jackson Labs) mice at 6-8 weeks of age and 20 – 23 g average body weight were used. The mice were housed and maintained in laminar flow rooms under specific pathogen-free conditions. All animal procedures were performed according to the guidelines of the Canadian Council on Animal Care (CACC). The protocol for animal studies was approved by the Animal Care Committee of the University of Ottawa.

Briefly, 786-O control and *RB1* KO cells were harvested at exponential growth phase using 0.25% trypsin-EDTA (25200-056, Thermo Fisher Scientific). Cells were washed once and resuspended in PBS. The number of viable cells was determined by trypan blue exclusion assay. 8 million viable cells were resuspended in 50% (vol/vol) Matrigel to a final volume of 100 µL and injected subcutaneously into the left or right flank of each mouse. Mice were randomly allocated to control/*RB1* KO or *RB1* KO/*RB1* KO groups. 5 control injections and 10 *RB1* KO injections were performed. The injection sites were manually palpated twice weekly until tumors were established. Caliper measurements of the length and width of the tumor were taken weekly until the onset of tumor growth and then twice weekly until endpoint. Mice weights were recorded every 3 days. All mice were euthanized at 11 weeks post-injection when the first mouse had reached endpoint. Tumors from each group were excised and weighed. Tumor measurements were performed by a collaborator who was blinded to the expected results of the study.

### Immunohistochemistry (IHC) and hematoxylin-eosin (HE) staining

Formalin-fixed tumor tissues were embedded in paraffin prior to IHC and HE staining. Cores were obtained from various parts of the tumors and 4 μm thick sections used to create a tumor microarray (TMA), containing control and *RB1* KO tumors. Before staining, the sections were deparaffinized and rehydrated, followed by antigen-retrieval in citrate buffer (pH 6) or Tris-EDTA (pH 9) as appropriate for 20 minutes. Tissue sections were blocked for 30 minutes using 10% goat serum (ab7481, Abcam), then incubated with primary antibody, followed by HRP-conjugated secondary antibody. Slides were stained using 3, 3’-diaminobenzidine (DAB) as the chromogen, counterstained with hematoxylin, mounted and cover slipped. The staining intensity was scored as follows: 0 (no staining), 1 (weak staining), 2 (moderate staining) and 3 (strong staining). The proportion of positively stained cells was scored according to the following criteria: 0 ( < 10% positive cells), 1 (10–25% positive cells), 2 (26–50% positive cells), 3 (51–75% positive cells) and 4 ( > 75% positive cells). The final staining score was calculated as the staining intensity score × the proportion score and ranged from 0 to 12. The following antibodies were used for IHC staining: pRb (554136, BD Biosciences) and SKIDA1 (BS-9776R, Thermo Fisher Scientific).

### CRISPR/Cas9 genome editing

Monoclonal 786-O *RB1* KO cell lines were generated with CRISPR/Cas9 technology. 786-O cells were transfected with PX458 plasmid (#48138, Addgene) containing *RB1*-targeting single guide RNAs (sgRNAs). Transfected cells were selected by single-cell sorting of green fluorescent protein (GFP)-expressing cells. Monoclonal cells were screened for target gene knockout by western blot and later, Sanger sequencing of the region of interest. Successful knockout clones were then selected and expanded.

The following guide RNAs were used to create monoclonal *RB1* knockout cell lines: *RB1* sgRNA 1 – CGGTGGCGGCCGTTTTTCGG; *RB1* sgRNA 2 – GCTCTCTCTCTGACATGATC. Polyclonal knockout cell lines were generated by stable expression of Cas9 (#52962, Addgene) in target cells, followed by transduction with lentivirus containing the corresponding guide RNAs in the pCLIP-dual-SFFV-ZsGreen vector backbone. Each pCLIP-dual-SFFV-ZsGreen vector contains 2 guides (gRNA_a and gRNA_b) targeting the gene of interest. All guides were designed against human genes. pCLIP-dual-SFFV-ZsGreen sgRNAs were obtained from transEDIT-dual CRISPR Whole Genome Arrayed Library from Transomic technologies. Target sequences were as follows: *SKIDA1* #1 (**gRNA_a:** GAAAATAAGCAGGGTCCGAG; **gRNA_b:** AATCCACTGGCTCAAAGTCA), *SKIDA1* #2 (**gRNA_a:** ACAACAAAGAATACTCCGAG; **gRNA_b:** GCAACTCCTCCAGATCGCAG), *E2F11* #1 (**gRNA_a:** GCAGCAGGTCAGGGTCGCAG; **gRNA_b:** CCACAGGTGTGAAATCCCCG), *E2F1* #2 (**gRNA_a:** CAAGCCCTGTCAGAAATCCA; **gRNA_b:** GGGCAGCCTGCGGGCTCCCG).

### Plasmids and siRNA constructs

HA-VHL wt-pBabe-puro (#19234, Addgene) plasmid was used for transient transfections. HA-VHL-pRc/CMV (#19999, Addgene) plasmid was used to create stable cell lines. Flag-pRb plasmid was obtained by cloning Flag-tagged pRb into the pLVX-M-puro vector (#125839, Addgene). For overexpression studies, the gene of interest was cloned into the pLVX-M-puro vector using Gibson cloning. All plasmids were sequenced to confirm validity. ON-TARGETplus Non-targeting Control siRNAs (D-001810-01-05) and siGENOME Human RB1 siRNA smartpool (M-003296-03-0005) were purchased from Dharmacon.

### Statistical analysis

Statistical analyses were performed using the GraphPad Prism 6 software. Data are presented as mean ± standard deviation (SD) unless otherwise indicated. Statistical significance was evaluated by unpaired two-tailed t-test, ordinary one-way ANOVA or two-way ANOVA with Holm-Sidak’s post-hoc test or Tukey’s post-hoc test as described in figure legends. p-values < 0.05 were considered statistically significant. Statistical significance was represented as: *p < 0.05, **p < 0.01, and ***p < 0.001. ‘ns’ denotes not significant. Experiments were performed in at least three biological replicates in order to establish statistical significance (unless otherwise stated). Number of replicates are provided in the individual figure legends.

## Supplementary information

Supplementary material

Supp Tables

Reproducibility Checklist

Uncropped western blots

## Data Availability

RNA-Seq data are deposited in GEO (GSE293447). All other datasets used and/or analyzed during the current study are available from the corresponding author upon reasonable request.
