# Supplementary material for: Loss of VHL-mediated pRb regulation promotes clear cell renal cell carcinoma
Source: Cell Death Dis. 2025 Apr 16;16(1):307. doi: 10.1038/s41419-025-07623-y (PMC12003641; doi:10.1038/s41419-025-07623-y)
Supplement: Supplementary file 1 — Supplementary material [file 41419_2025_7623_MOESM1_ESM.pdf]

SUPPLEMENTARY FIGURES

Loss of VHL-mediated pRb regulation promotes clear cell renal cell carcinoma.

Mercy Akuma[1], Minjun Kim [2,3†], Chenxuan Zhu [1†], Ellis Wiljer [4], Antoine Gaudreau-Lapierre[1], Leshan D. Patterson[5], Lars Egevad[6], Simon Tanguay[7], Laura Trinkle-Mulcahy [1], William L. Stanford[1,4,8,9], Yasser Riazalhosseini [2,3], Ryan C. Russell[*1,9,10].

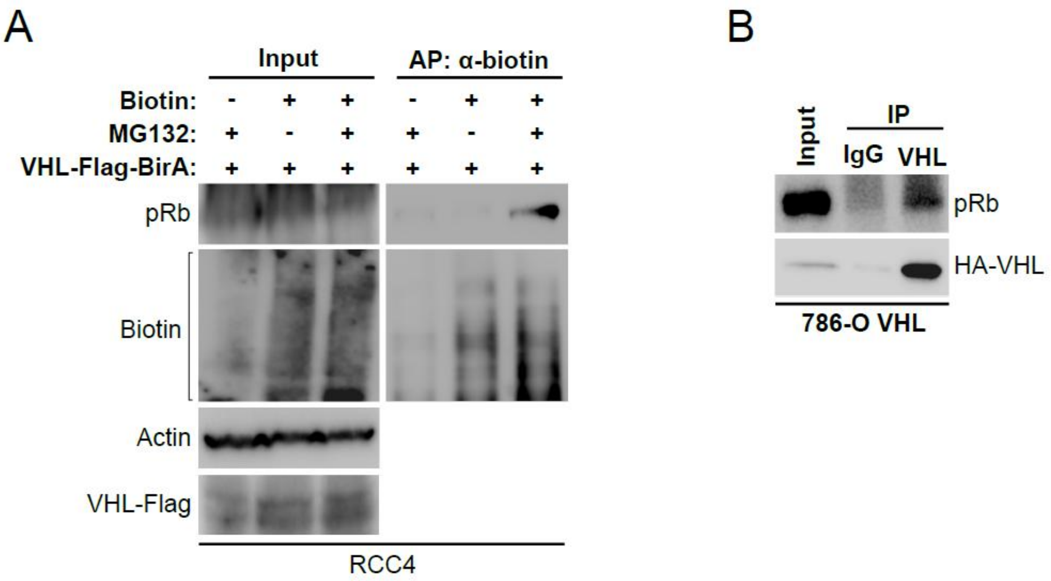

Supplementary Figure S1. (A) Affinity purification of biotinylated proteins using streptavidin-conjugated beads from RCC4 cells transfected with VHL-Flag-BirA plasmid and treated with 10 µM MG132 and 50 µM biotin as indicated for 4 h and 1 h respectively. **(B)** Immunoprecipitation of VHL from 786-O cells stably transfected with hemagglutinin (HA)-tagged VHL and treated with 10 µM MG132 for 4 h. An IgG1 isotype control was included. MG132-treated lysate was split equally between IgG1 and VHL pulldown conditions.

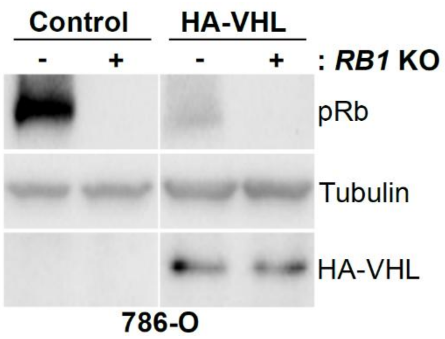

Supplementary Figure S2. Immunoblot of lysates from 786-O control and *RB1* KO cells stably transfected with either control vector or hemagglutinin (HA)-tagged VHL.

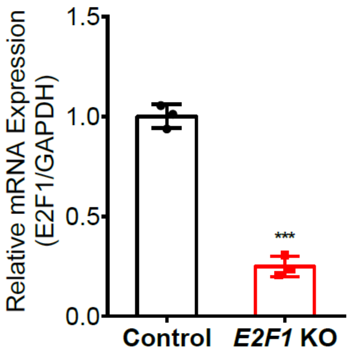

Supplementary Figure S3. E2F1 mRNA expression (relative to GAPDH) in 786-O Cas9-expressing cells stably infected with virus encoding either control vector or *E2F1*-targeting guides. Fold changes in gene expression were calculated using the delta delta Ct method. Statistical significance was calculated using unpaired two-tailed t test (n=3). Data are represented as mean ± SD. *** p < 0.001.

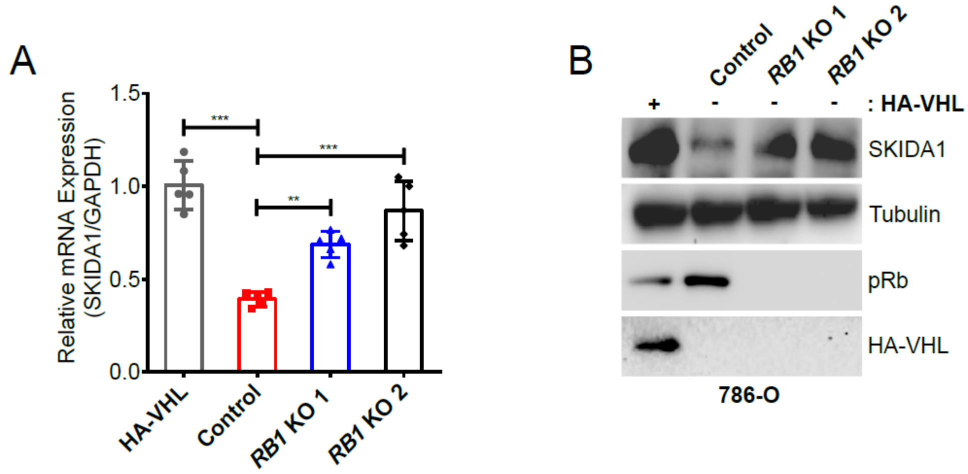

Supplementary Figure S4. (A) SKIDA1 mRNA expression (relative to GAPDH) in 786-O control, VHL-expressing and *RB1* KO cells. Fold changes in gene expression were calculated using the delta delta Ct method and normalized to the control condition. Statistical significance was calculated using ordinary one-way ANOVA and Tukey's post-hoc test (n=5). Data are represented as mean ± SD. **$p < 0.01$, ***$p < 0.001$. **(B)** Immunoblot of lysates from 786-O control, VHL-expressing and *RB1* KO cells.

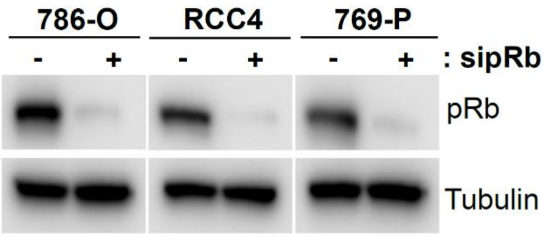

Supplementary Figure S5. Immunoblot of lysates from indicated ccRCC cell lines transfected with either non-targeting control siRNA (siCtrl) or pRb targeting siRNA (sipRb).

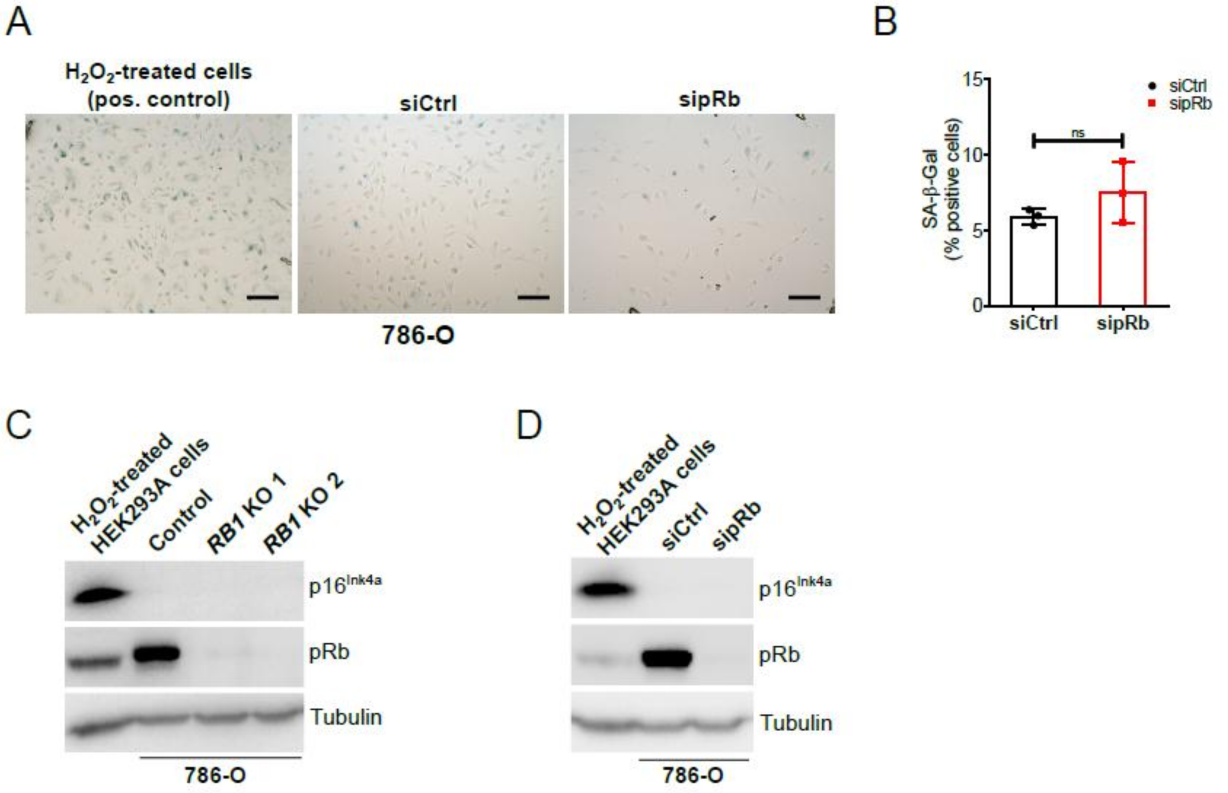

Supplementary Figure S6. (A) Representative images showing beta-galactosidase staining of 786-O cells transfected with either non-targeting control siRNA (siCtrl) or pRb-targeting siRNA (sipRb). Positive control cells were obtained by treating 786-O cells with 150 µM hydrogen peroxide (H_2O_2) for 2 hours, followed by recovery for five days. Scale bar = 250 µm. **(B)** Quantification of SA-β-gal positive cells in (A). Statistical significance was calculated using unpaired two-tailed t test (n=3). Data are represented as mean ± SD. ns denotes not significant. **(C)** Immunoblot analysis of $p16^{Ink4a}$ expression in 786-O control and *RB1* KO cells. HEK293A cells treated with 150 µM H_2O_2 for five days, were used as positive control for senescence induction and $p16^{Ink4a}$ expression. **(D)** Immunoblot analysis of p16Ink4a expression in 786-O cells transfected with either non-targeting control siRNA (siCtrl) or pRb-targeting siRNA (sipRb). HEK293A cells treated with 150 µM H_2O_2 for five days, were used as positive control for senescence induction and $p16^{Ink4a}$ expression.

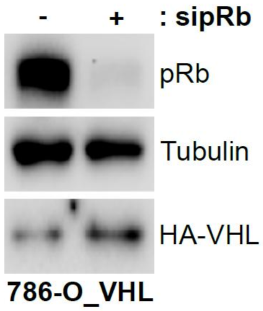

Supplementary Figure S7. Immunoblot of lysates from VHL-expressing 786-O cells transfected with either non-targeting control siRNA (siCtrl) or pRb targeting siRNA (sipRb).

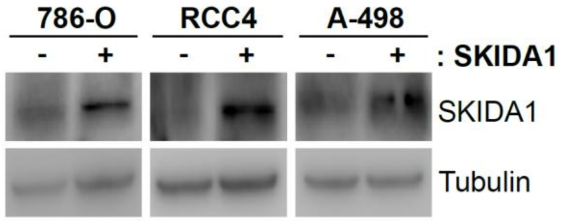

Supplementary Figure S8. Immunoblot of lysates from 786-O, RCC4 and A-498 cells stably infected with lentivirus encoding either control plasmid (-) or SKIDA1 cDNA containing plasmid.

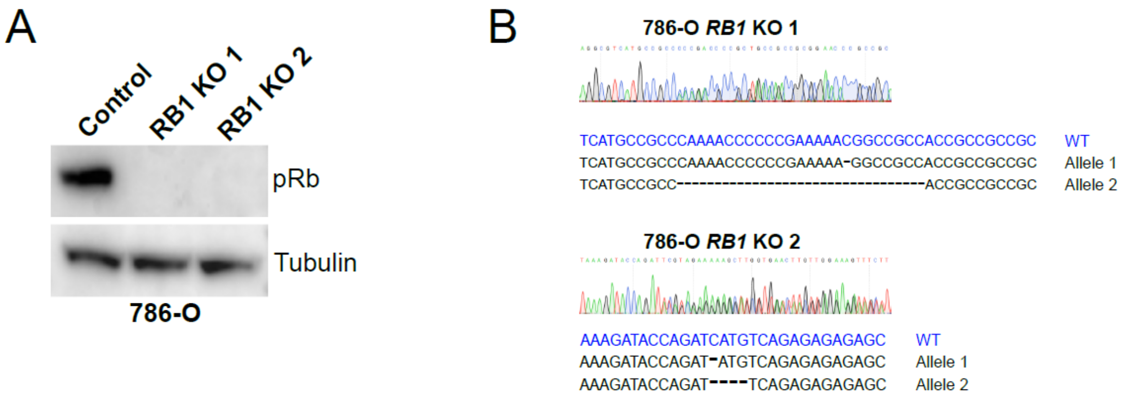

Supplementary Figure S9. (A) Immunoblot of lysates from 786-O control and *RB1* KO cells. **(B)** Sanger sequencing of 786-O *RB1* KO clones and sequence alignment against wild-type at the *RB1* locus.

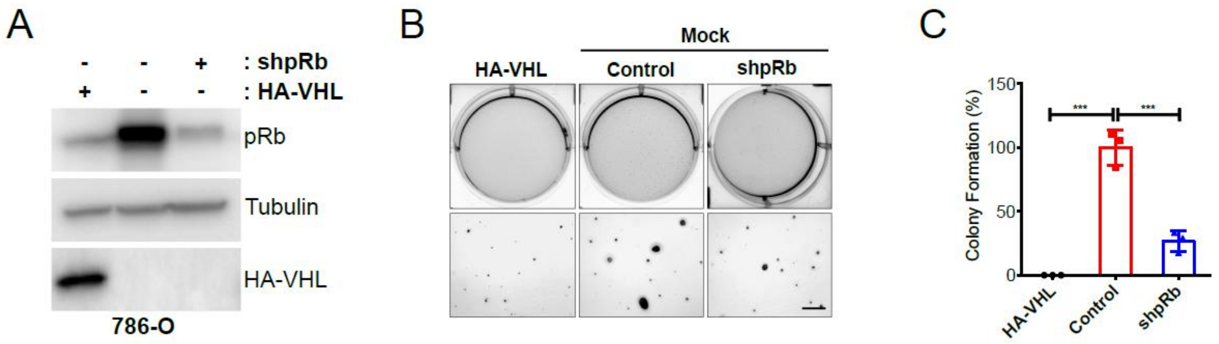

Supplementary Figure S10. (A) Immunoblot of lysates from 786-O control, VHL-reconstituted, and pRb knockdown cells. For VHL reconstitution, 786-O cells were stably transfected with hemagglutinin (HA)-tagged VHL. pRb knockdown cells were made by infecting 786-O cells with lentivirus encoding pRb-targeting shRNA. **(B)** Representative images showing colony formation in soft agar of 786-O control, VHL-reconstituted, and pRb knockdown (shpRb) cells. Scale bar = 250 µm. **(C)** Quantification (relative to control) of the number of colonies formed in (B). Colonies were manually counted under a light microscope. Statistical significance was calculated using ordinary one-way ANOVA and Tukey's post-hoc test (n=3). Data are represented as mean ± SD. *** $p < 0.001$.

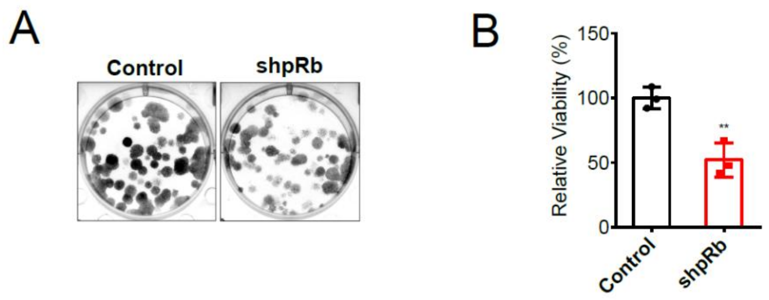

Supplementary Figure S11. (A) Representative images showing clonogenic outgrowth of 786-O control and pRb knockdown cells. **(B)** Quantification (relative to control) of the number of colonies in (A). Statistical significance was calculated using unpaired two-tailed t test (n=3). Data are represented as mean ± SD. ** $p < 0.01$.

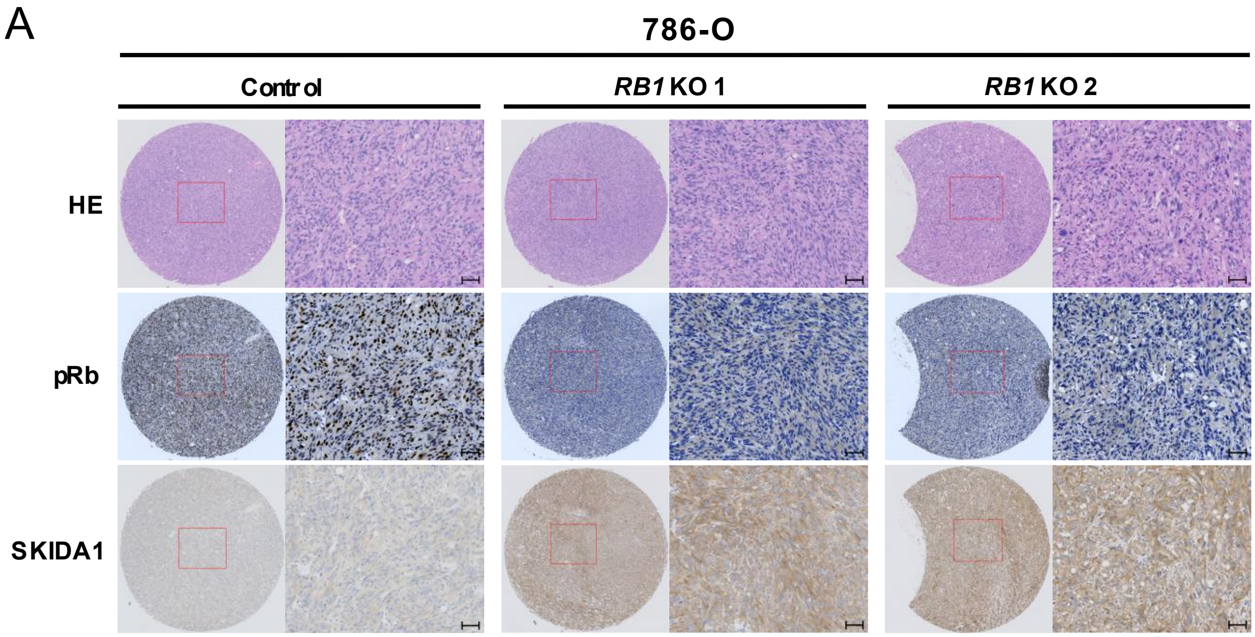

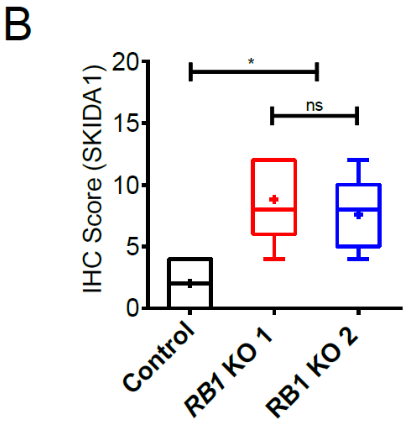

Supplementary Figure S12. (A) Representative images showing HE and IHC staining of the indicated proteins on tumor sections from mice xenograft injections of 786-O control or *RB1* KO cells. Scale bar = 50 µm. **(B)** Box and whisker plot showing semi-quantitative IHC scoring of SKIDA1 protein expression in tumors formed by 786-O control and *RB1* KO cells injected subcutaneously into the flanks of immunodeficient *NOD-scid IL2Rg*^{null} mice, via a tumor xenograft assay. Mean is shown as '+'. Statistical significance was calculated using ordinary one-way ANOVA and Tukey's post-hoc test (n>3). One control injection which failed to grow was considered an outlier and omitted during statistical analysis.

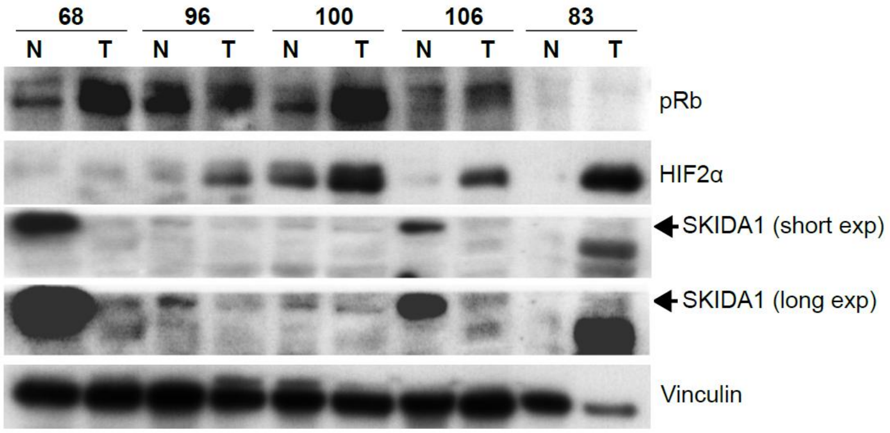

Supplementary Figure S13. Immunoblot of lysates from ccRCC primary tumor (T) and patient-matched normal tissue (N). All tumors were genotyped and confirmed to have VHL mutations.
