# Supplementary figures and images for: Loss of VHL-mediated pRb regulation promotes clear cell renal cell carcinoma
Source: Cell Death Dis. 2025 Apr 16;16(1):307. doi: 10.1038/s41419-025-07623-y (PMC12003641; doi:10.1038/s41419-025-07623-y)

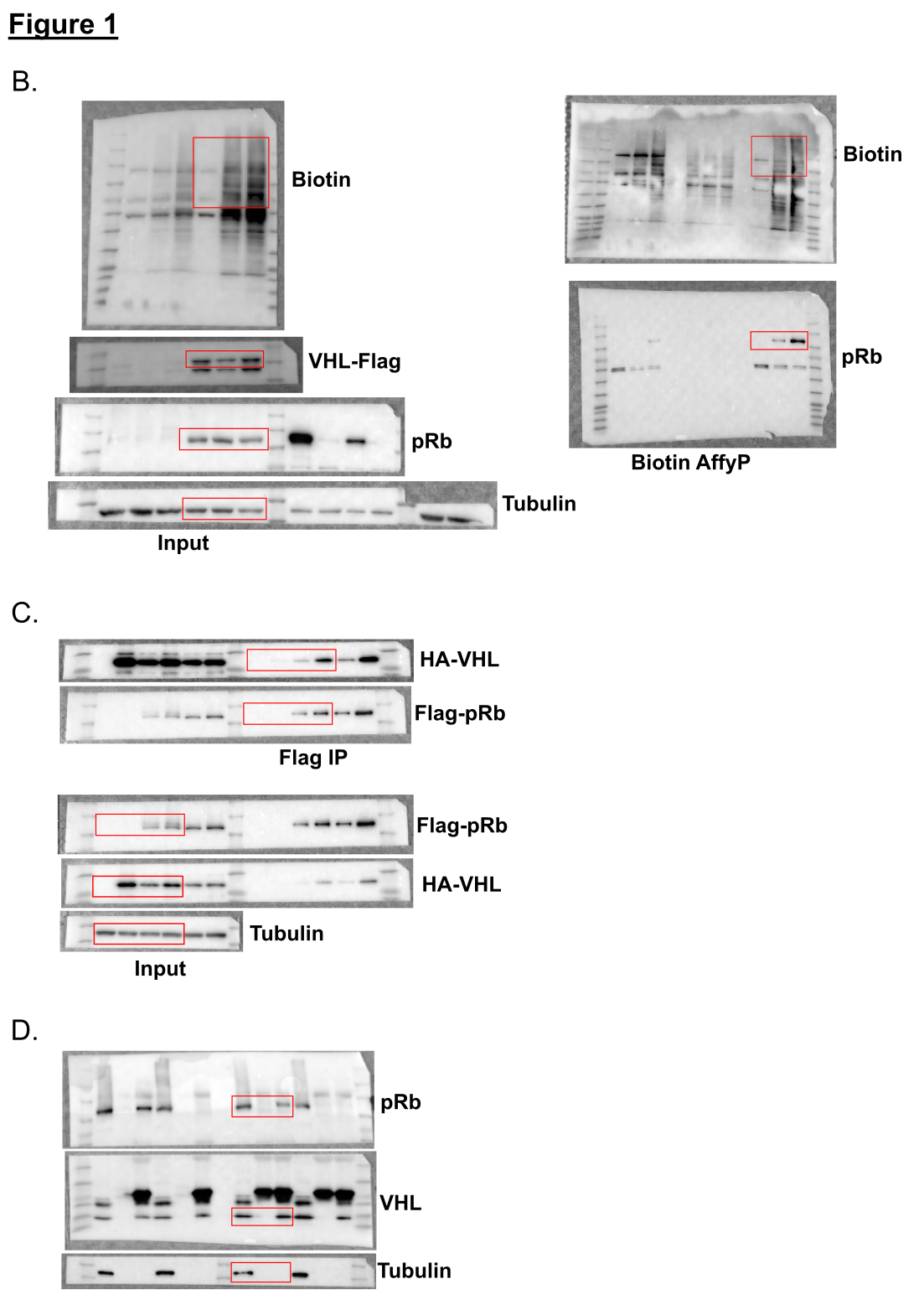


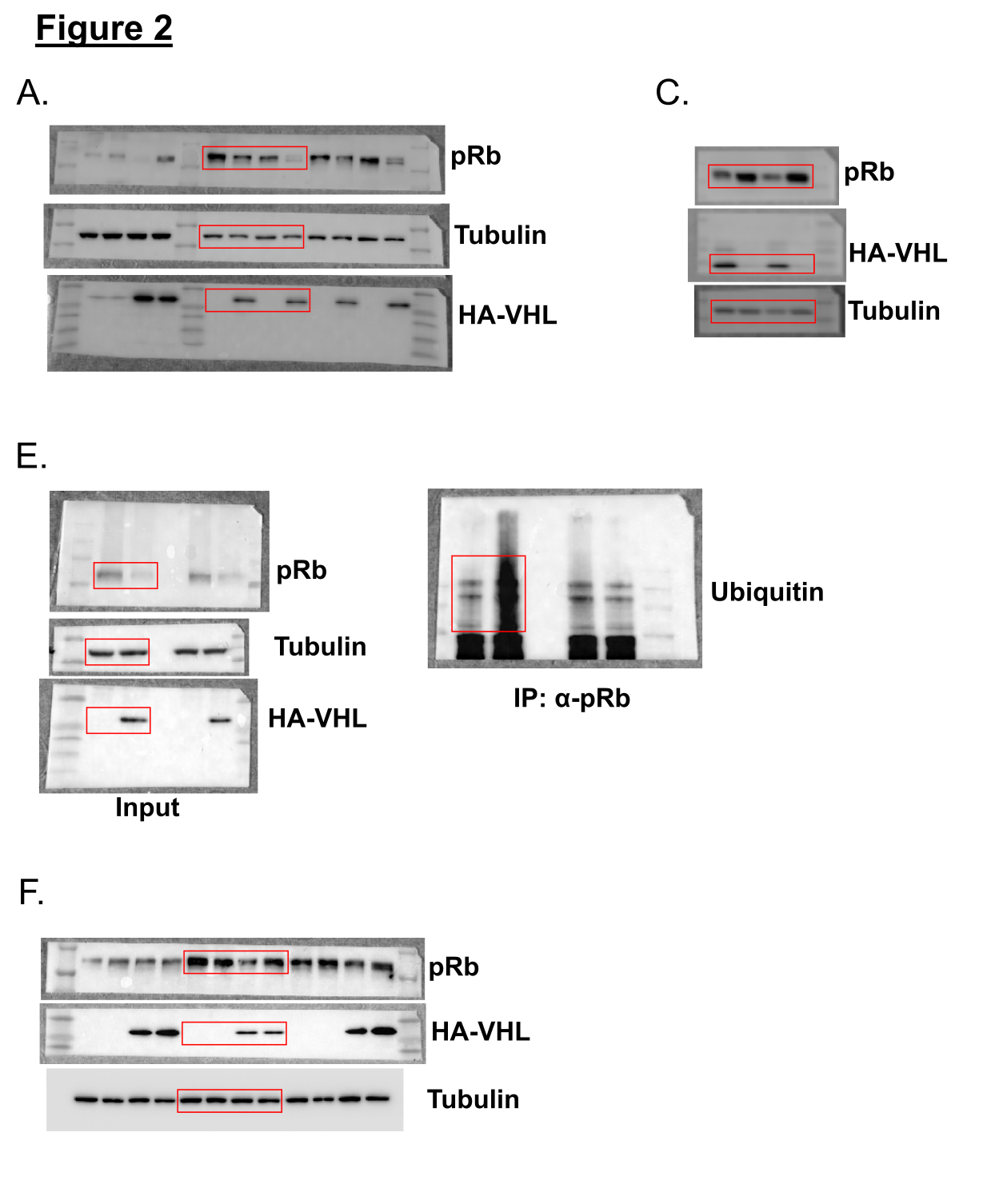


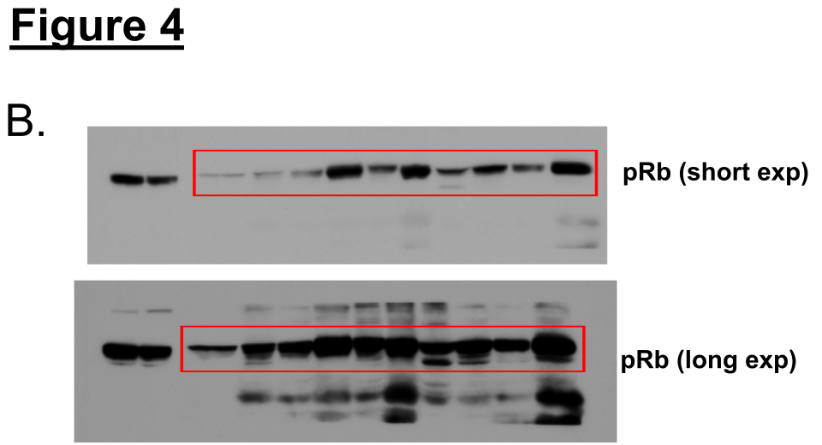

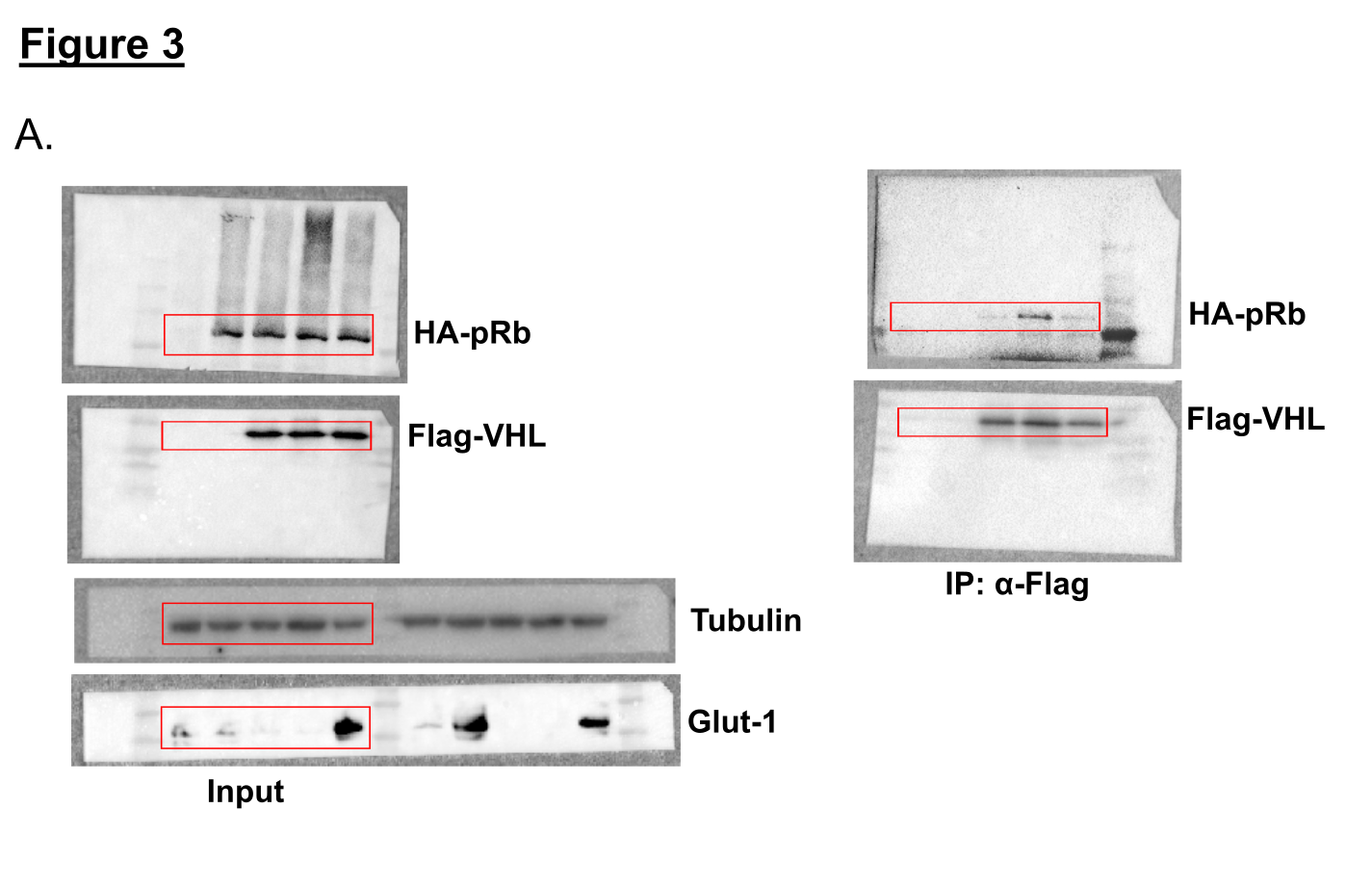


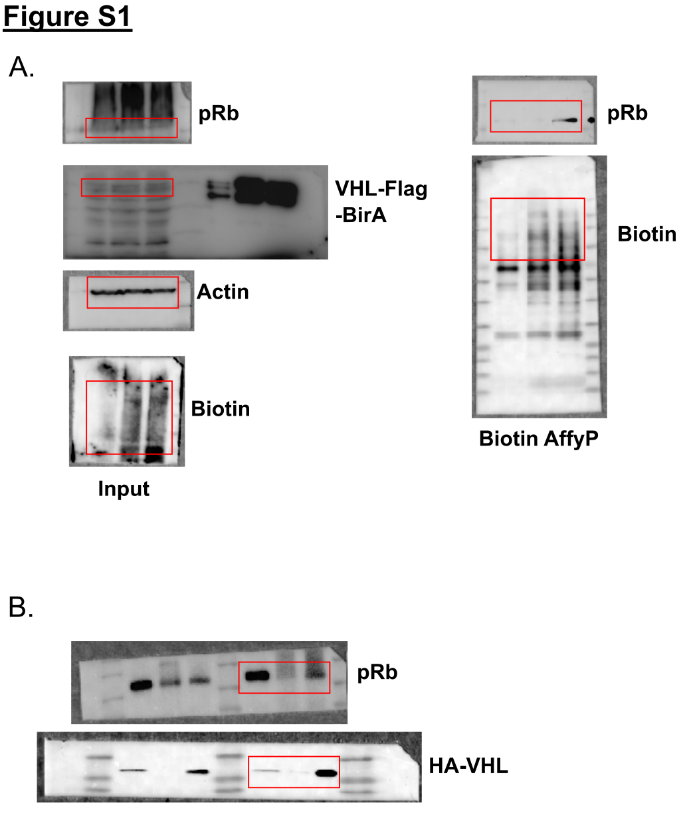


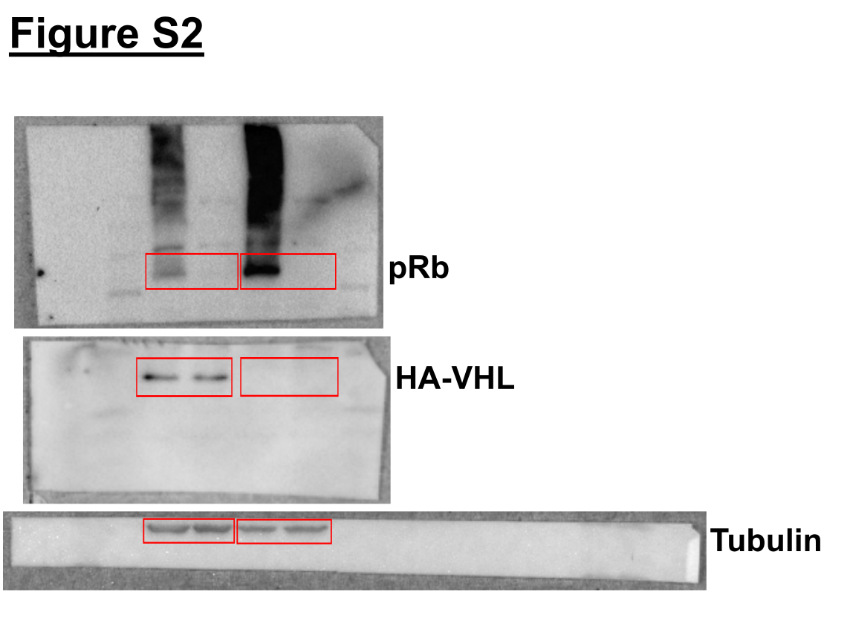


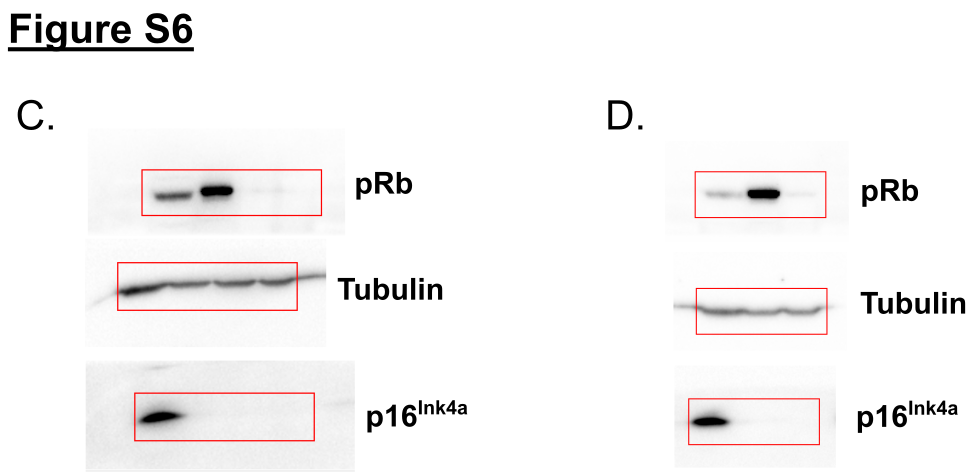

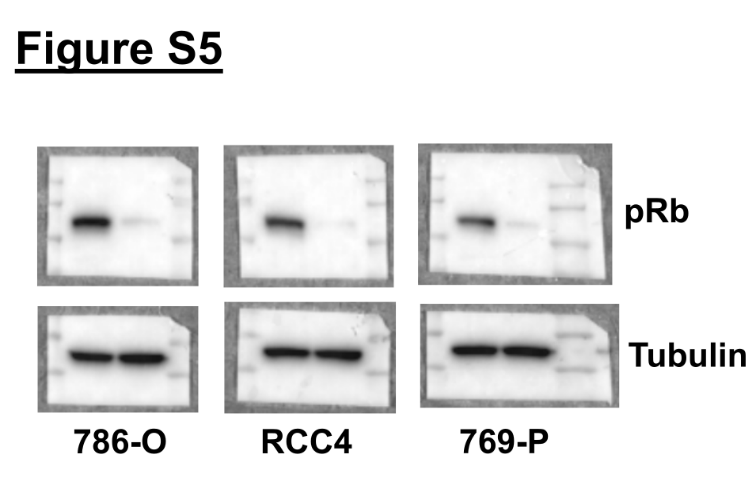

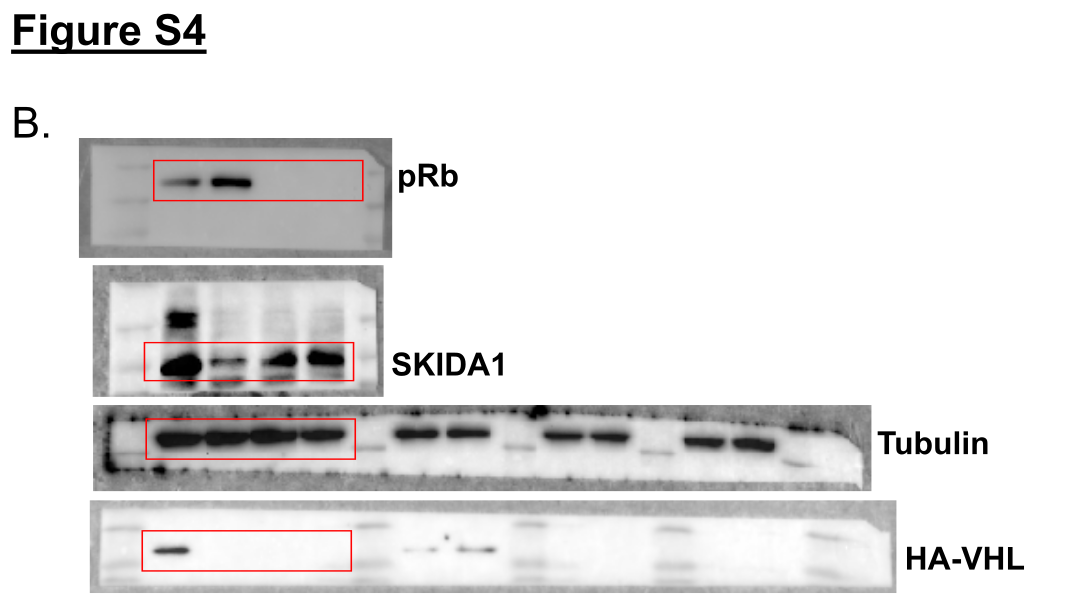


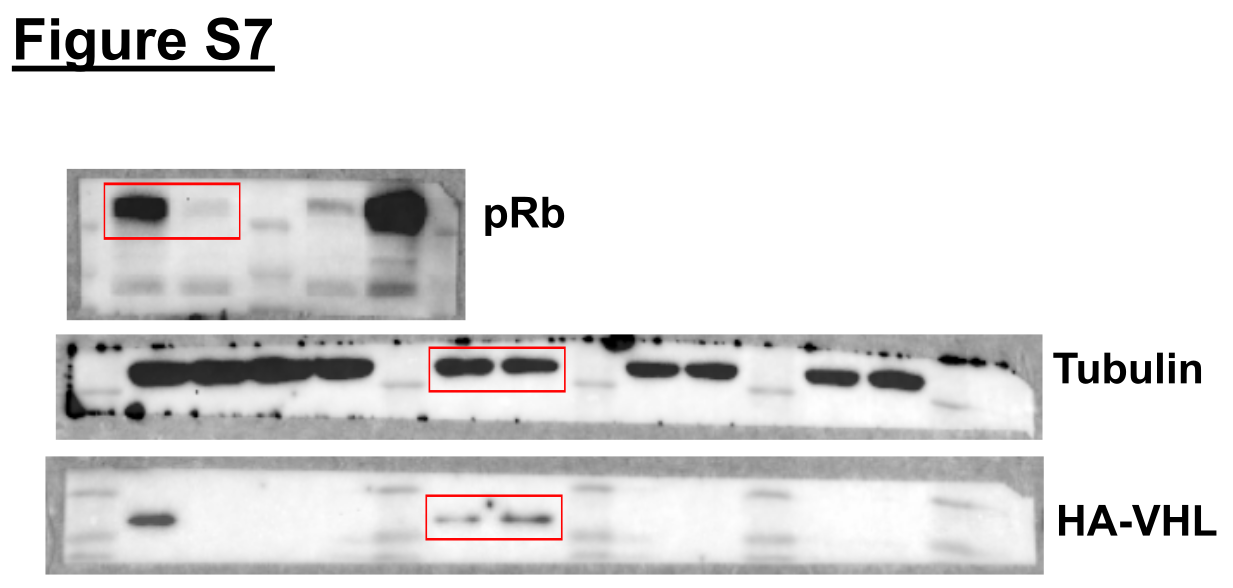


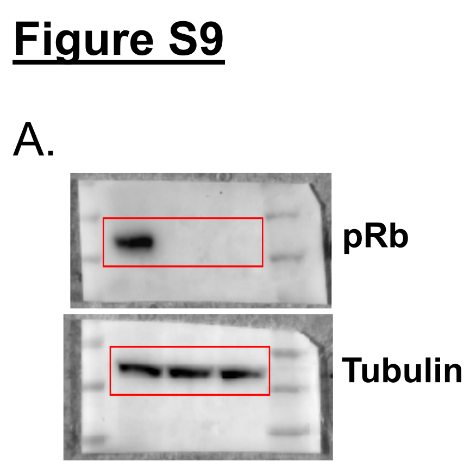

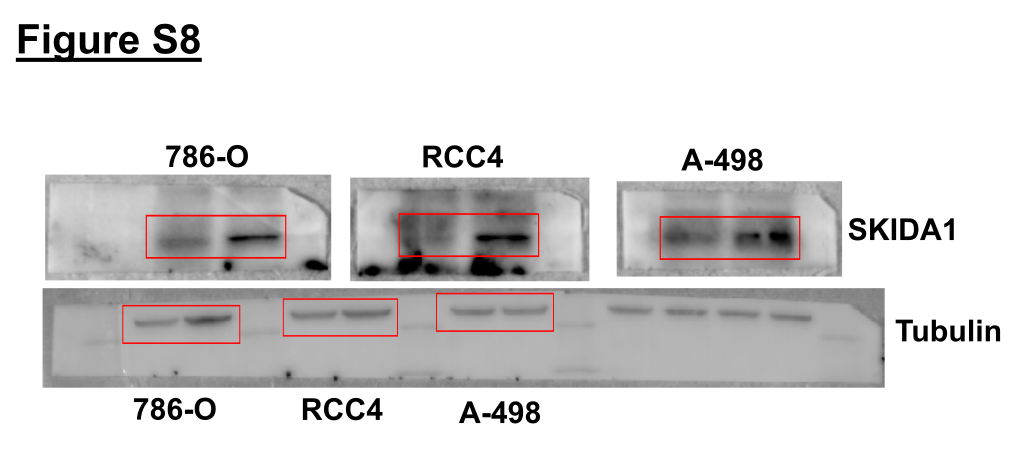


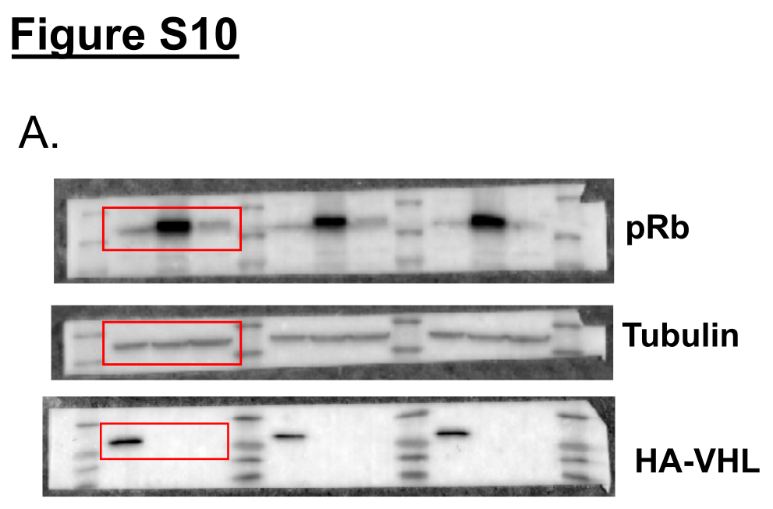


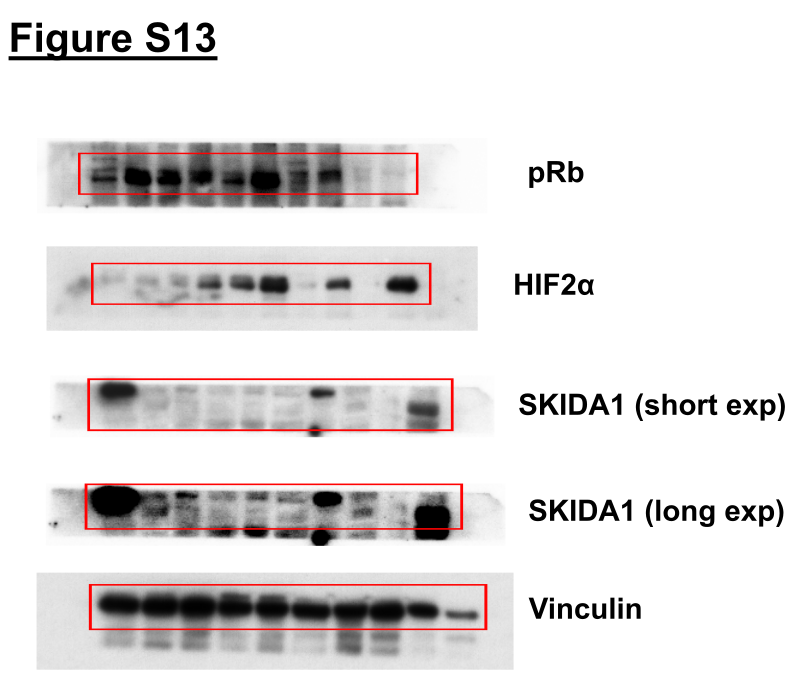

Supplement: Supplementary file 4 — Uncropped western blots [file 41419_2025_7623_MOESM4_ESM.docx]
